# Transcriptome characterization of BPG axis and expression profiles of ovarian steroidogenesis-related genes in the Japanese sardine

**DOI:** 10.1186/s12864-020-07080-1

**Published:** 2020-09-29

**Authors:** Mitsuo Nyuji, Yuki Hongo, Michio Yoneda, Masahiro Nakamura

**Affiliations:** 1 Fisheries Resources Institute, Japan Fisheries Research and Education Agency, Yokohama, 236-8648 Japan; 2 Hakatajima Field Station, Fisheries Technology Institute, Japan Fisheries Research and Education Agency, Kinoura, Imabari, Ehime 794-2305 Japan

**Keywords:** Clupeoid fish, RNA-seq, Reproduction, Ovary, Fsh, Lh, Steroidogenic enzyme

## Abstract

**Background:**

The clupeoid fishes are ecologically and commercially important fish species worldwide that exhibit a high level of population fluctuation, accompanied by alteration of reproductive traits. However, knowledge about their reproductive physiology in order to understand mechanisms underlying such population dynamics is limited. The endocrine system along with the brain–pituitary–gonadal (BPG) axis is critical for regulating reproduction. The aims of this study were to provide transcript data and genes related to the BPG axis, and to characterize the expression profiles of ovarian steroidogenesis-related genes in the Japanese sardine (*Sardinops melanostictus*, Clupeidae).

**Results:**

RNA sequencing was performed using the sardine brain, pituitary, and gonad in both sexes. A total of 290,119 contigs were obtained and 115,173 non-redundant ORFs were annotated. The genes differentially expressed between ovary and testis were strongly associated with GO terms related to gamete production. The tissue-specific profile of the abundance of transcripts was characterized for the major regulators in the BPG axis, such as gonadotropin-releasing hormone, gonadotropin, and steroidogenic enzyme. By comparing between ovary and testis, out of eight different 17β-hydroxysteroid dehydrogenase (Hsd17b) genes identified, higher *hsd17b7* expression was found in testis, whereas higher expression of *hsd17b8*, *hsd17b10*, *hsd17b12a*, and *hsd17b12b* was found in ovary. The cDNAs encoding key endocrine factors in the ovarian steroidogenic pathway were cloned, sequenced, and quantitatively assayed. In the pituitary, *follicle-stimulating hormone beta* peaked during vitellogenesis, while *luteinizing hormone beta* peaked at the completion of vitellogenesis. In the ovary, *follicle-stimulating hormone receptor* and *luteinizing hormone receptor* were upregulated from mid- to late phase of vitellogenesis. Furthermore, three steroidogenic enzyme genes (*cyp11a1*, *cyp17a1*, and *cyp19a1a*) gradually increased their expression during ovarian development, accompanying a rise in serum estradiol-17β, while *3β-hydroxysteroid dehydrogenase* and *steroidogenic acute regulatory protein* did not change significantly.

**Conclusions:**

This is the first report of deep RNA sequencing analysis of Japanese sardine, in which many key genes involved in the BPG axis were identified. Expression profiles of ovarian steroidogenesis-related genes provide a molecular basis of the physiological processes underlying ovarian development in the sardine. Our study will be a valuable resource for clarifying the molecular biology of clupeoid fishes.

## Background

The application of next-generation sequencing (NGS) technology has recently increased in the field of aquaculture and fisheries. For aquaculture fish species, the administration of exogenous hormones and environmental manipulation are conducted to control gonadal development in captivity. Since knowledge of reproductive physiology is helpful to develop effective treatments for such control, RNA sequencing (RNA-seq) has been applied for a variety of aquaculture species to reveal the physiological mechanisms regulating reproduction. As recent examples, RNA-seq analyses were conducted on carp, eel, perch, and salmonid species [1–5]. For important target species of fisheries, the application of large-scale genomic approaches has mostly been conducted to investigate population structure and genetic differentiation (e.g., for cod [6] and tuna species [7]). Accordingly, the current understanding of large-scale transcriptome data for fish reproduction is considered to be biased towards aquaculture species. Meanwhile, recent research on swordfish (*Xiphias gladius*) fisheries showed that RNA-seq analysis of ovarian development can be a useful tool in establishing a biological basis of reproduction for successful stock management [8].

Clupeoid fishes include ecologically and commercially important fish species, such as anchovies, herrings, and sardines. They exhibit dramatic and cyclic population dynamics in response to climate change at the multi-decadal scale [9, 10]. Since such population fluctuation is accompanied by alteration of reproductive traits [11], knowledge about the reproductive physiology is key to understanding the mechanisms of such population dynamics. The first draft genome of Atlantic herring (*Clupea harengus*) was published in 2016 by Martinez Barrio et al. [12] and the genome assembly has recently been improved by Kongsstovu et al. [13]; however, gene profiling related to reproduction has not been characterized.

As in other vertebrates, fish reproduction is regulated by the activation of endocrine factors in the brain–pituitary–gonadal (BPG) axis. The brain gonadotropin-releasing hormone (Gnrh) stimulates the secretion of pituitary gonadotropins (Gths), follicle-stimulating hormone (Fsh) and luteinizing hormone (Lh), which in turn act on the gonads to stimulate the production of sex steroid hormones, such as estrogens and androgens [14–16]. The Gnrhs are decapeptides, of which teleost fish commonly have two or three forms within the brain of individual species, that is, species-specific-type Gnrh (Gnrh1), chicken-II-type Gnrh (Gnrh2), and salmon-type Gnrh (Gnrh3) [17]. The Gths are glycoprotein hormones composed of a common glycoprotein α-subunit (Cga) and hormone-specific β-subunit (Fshb or Lhb) [18].

During oogenesis, the substantial growth of the oocyte is marked by vitellogenesis (yolk protein accumulation), which occurs after previtellogenic growth with the appearance and accumulation of cortical alveoli [19]. The progress of vitellogenesis is generally accompanied by a rapid elevation of circulating estradiol-17β (E2), which induces hepatic vitellogenin production [19]. E2 is produced in oocyte-surrounding follicle cells which are composed of an inner layer of granulosa cells, a basement membrane, and an outer layer of thecal cells [19]. In the follicle cells, the physiological effects of Gths are mediated by Gth receptors (Gthrs; Fshr and Lhr) [20]. In the ovarian steroidogenic pathway, a series of enzymes, such as cytochrome P450 side-chain cleavage (*cyp11a1*), 17α-hydroxylase/17,20-lyase (*cyp17a1*), ovarian aromatase (*cyp19a1a*), and 3β-hydroxysteroid dehydrogenase (*hsd3b*), as well as steroidogenic acute regulatory protein (*star*), catalyze the conversion of cholesterol to E2 [19]. Information on the molecular regulation of steroidogenic proteins (steroidogenic enzymes and Star) in the ovarian steroidogenic pathway is a prerequisite for understanding physiological mechanisms governing ovarian development. In clupeoid fishes, there is no information available on the expression of genes encoding ovarian steroidogenic proteins.

The objective of the present study was to provide transcript data and genes related to the BPG axis with the goal of establishing a molecular basis for the endocrine regulation of ovarian development using the Japanese sardine (*Sardinops melanostictus*, Clupeidae). The Japanese sardine has been a key species for studies of population dynamics because of its high level of population fluctuation [21]. Although some evidence of the mechanisms of population dynamics has been presented from an ecological perspective [21–25], the available information on aspects of the endocrine regulation of ovarian development is still limited. RNA-seq was performed using sardine brain, pituitary, and gonad from both sexes to generate a high-quality transcriptome assembly. Furthermore, complementary DNAs (cDNAs) encoding key endocrine factors in the ovarian steroidogenic pathway were cloned and sequenced. We then analyzed their expression profiles during ovarian development by quantitative real-time PCR.

## Results

### Assembly and functional annotation

Japanese sardines at age 3 (three fish for each sex) sampled in February were used for RNA-seq. The body size was 186–203 mm in body length (BL) and 91–112 g in body weight (BW). Females had vitellogenic ovaries, while males were at the spermiation stage.

**Fig. 1:**
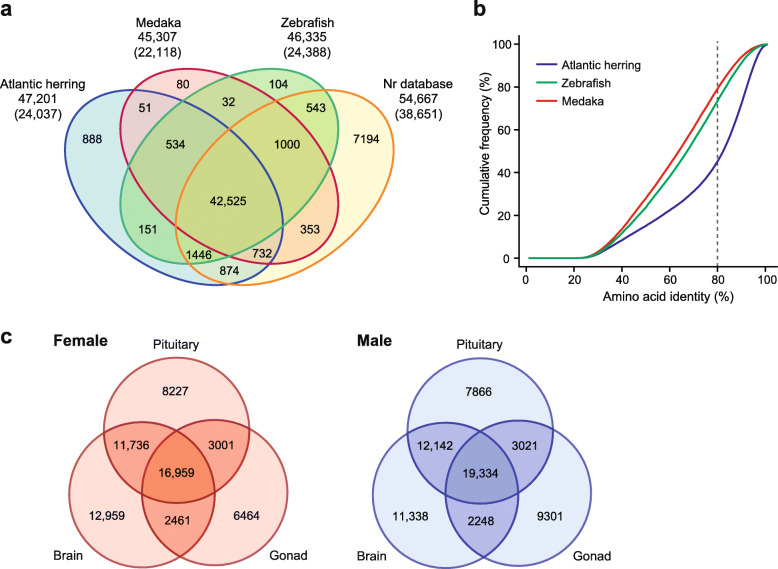
**a** Venn diagram showing the numbers of shared and unique protein sequences in the Japanese sardine (*Sardinops melanostictus*) relative to Atlantic herring (*Clupea harengus*), zebrafish (*Danio rerio*), medaka (*Oryzias latipes*), and the NCBI non-redundant protein (nr) databases. Total numbers of sardine proteins having similarity to each database are indicated under the database names. The parentheses indicate the number of unique accessions having similarity in the database. **b** Cumulative relative frequency distribution of amino acid sequence identity against Atlantic herring, zebrafish, and medaka. Dashed line indicates 80% amino acid identity. **c** Venn diagram showing the number of transcripts commonly and differentially expressed in the three tissues (brain, pituitary, and gonad) for female and male. The number of transcripts was counted when fragments per kilobase of transcript per million mapped read (FPKM) of each replicate was more than 1

Sequencing of the cDNA libraries using NextSeq500 yielded a total of 5–7 million pared-end reads (150 bp) per organ, which were deposited in the DNA Data Bank of Japan (DDBJ) Sequence Read Archive (DRA) under accession numbers DRA010129. The adapter and low-quality sequences were trimmed, and then the remaining reads from all libraries were assembled by Trinity into 290,119 contigs with an average length of 696 bp (Table 1). Open reading frames (ORFs) of more than 300 bp were extracted from the contigs and then the redundant protein sequences with up to 95% similarity were clustered into 115,173 distinct ORFs, which consisted of complete (47,886), internal (24,524), 5′ partial (30,603), and 3′ partial (12,160) ORFs (Table 1). Those translated ORFs were then used for sequence homology searches. Among a total of 115,173 protein sequences, 47,201 (41.0%), 46,335 (40.2%), 45,307 (39.3%), and 54,667 (47.5%) protein sequences showed significant similarities (e-value ≤1e−05) to protein sequences of Atlantic herring, zebrafish (*Danio rerio*), medaka (*Oryzias latipes*), and the NCBI non-redundant protein (nr) database, respectively (Fig. 1a). Of these, 24,037, 24,388, 22,118, and 38,651 protein sequences are allocated unique accession numbers for Atlantic herring, zebrafish, medaka, and the NCBI nr database, respectively (Fig. 1a). Overall, 888, 104, 80, and 7194 protein sequences had unique similarity to Atlantic herring, zebrafish, medaka, and the NCBI nr database, respectively, and 42,525 protein sequences shared similarity among the three species and the NCBI nr database (Fig. 1a). The cumulative relative frequency distribution for amino acid identities between sardine and the three fish species showed that, at 80% amino acid identity, the frequencies were 47.2% (Atlantic herring), 75.5% (zebrafish), and 80.9% (medaka) (Fig. 1b).

**Table 1:** Assembly details of Japanese sardine (*Sardinops melanostictus*) brain–pituitary–gonadal (BPG) axis transcriptome

Assembly	
Number of contigs	290,119
Total bases (bp)	201,987,287
Longest contig length (bp)	18,202
Shortest contig length (bp)	203
Average of contig length (bp)	696
N50	1165
Extraction of ORFs	
Number of non-redundant ORFs	115,173
Complete ORFs	47,886
Internal ORFs	24,524
5′ partial ORFs	30,603
3′ partial ORFs	12,160
Homology search against below organisms	
Atlantic herring (*Clupea harengus*)	47,201^a^
Zebrafish (*Danio rerio*)	46,335^a^
Medaka (*Oryzias latipes*)	45,307^a^

^a^Number of protein sequences

Additionally, a total of 9053 Gene Ontology (GO) numbers were assigned to 19,722 protein sequences (17.1%), and 828 enzyme commission (EC) numbers were assigned to 4588 protein sequences (4.0%). All of the contigs were registered with DDBJ as Transcriptome Shotgun Assembly (TSA) sequences under accession numbers ICPT01000001–ICPT01290119. The results of the Protein Basic Local Alignment Search Tool (BLASTP) are shown in Additional file 1. The statistical distribution of GO-slim categories is shown in Additional file 2: Figure S1.

### Differentially expressed genes

The numbers of transcripts commonly and differentially expressed in the three tissues (brain, pituitary, and gonad) for female and male were analyzed (Fig. 1c). In both sexes, the transcripts with the highest tissue-specificity were found in the brain, and the highest overlap of transcripts between two tissues was found between brain and pituitary. The number of transcripts shared by all tissues was 16,959 in female, while it was 19,334 in male. The numbers of tissue-specific transcripts of brain and pituitary were similar between female and male, while in the gonad, this was approximately 1.4 times higher in testis than in ovary.

Comparative expression profiling between ovary and testis showed that 9746 and 15,804 transcripts were significantly expressed (false discovery rate: FDR < 0.05), and 61 and 33 GO terms were significantly enriched (FDR < 0.05) in ovary and testis, respectively (Additional file 3: Table S1 and S2). Among the top 10 GO terms enriched in the ovary, five germ cell-related terms, namely, positive regulation of acrosome reaction (GO:2000344), acrosin binding (GO:0032190), egg coat formation (GO:0035803), binding of sperm to zona pellucida (GO:0007339), and structural constituent of egg coat (GO:0035804), were found (Table 2). Among the top 10 GO terms enriched in the testis, spermatogenesis (GO:0007283) and six cilium-related terms, namely, ciliary basal body (GO:0036064), cilium movement (GO:0003341), intraciliary transport (GO:0042073), motile cilium (GO:0031514), ciliary transition zone (GO:0035869), and outer dynein arm assembly (GO:0036158) were found (Table 3).

**Table 2:** Top 10 enriched GO terms in the ovary from the genes differentially expressed between ovary and testis

GO ID	GO Term	GO Category	FDR	*P*-Value
GO:0016592	mediator complex	CC	5.23E-08	3.84E-11
GO:2000344	positive regulation of acrosome reaction	BP	1.97E-05	3.54E-08
GO:0032190	acrosin binding	MF	1.97E-05	3.54E-08
GO:0035803	egg coat formation	BP	2.22E-05	4.53E-08
GO:0007339	binding of sperm to zona pellucida	BP	2.22E-05	4.53E-08
GO:0031941	filamentous actin	CC	2.98E-04	1.09E-06
GO:0003712	transcription coregulator activity	MF	1.46E-03	6.68E-06
GO:0035804	structural constituent of egg coat	MF	1.65E-03	8.08E-06
GO:0006767	water-soluble vitamin metabolic process	BP	5.01E-03	3.72E-05
GO:1903047	mitotic cell cycle process	BP	5.24E-03	3.93E-05

*P*-values on the right are derived from Fisher’s exact test. *P*-values were adjusted by false discovery rate (FDR) presented in the column ‘FDR’. GO categories are biological process (BP), molecular function (MF), and cellular component (CC). For a complete list, see Additional file 3: Table S1

**Table 3:** Top 10 enriched GO terms in the testis from the genes differentially expressed between ovary and testis

GO ID	GO Term	GO Category	FDR	*P*-Value
GO:0036064	ciliary basal body	CC	3.79E-09	2.78E-12
GO:0003341	cilium movement	BP	7.37E-09	6.61E-12
GO:0042073	intraciliary transport	BP	9.39E-06	1.68E-08
GO:0031514	motile cilium	CC	3.61E-05	7.39E-08
GO:0007062	sister chromatid cohesion	BP	1.19E-04	3.01E-07
GO:0035869	ciliary transition zone	CC	1.29E-04	3.47E-07
GO:1990108	protein linear deubiquitination	BP	2.79E-04	8.86E-07
GO:0000795	synaptonemal complex	CC	2.79E-04	8.86E-07
GO:0007283	spermatogenesis	BP	3.30E-04	1.16E-06
GO:0036158	outer dynein arm assembly	BP	8.33E-04	3.19E-06

*P*-values on the right are derived from Fisher’s exact test. *P*-values were adjusted by false discovery rate (FDR) presented in the column ‘FDR’. GO categories are biological process (BP) and cellular component (CC). For a complete list, see Additional file 3: Table S2

### Identification of reproduction-related genes in the BPG axis

**Fig. 2:**
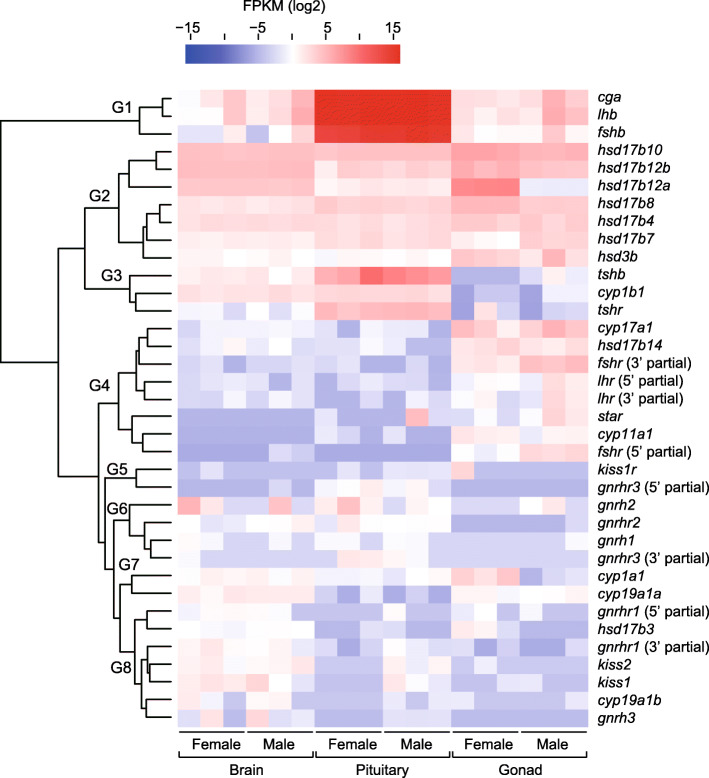
Heatmap of transcripts involved in endocrine regulation of the brain–pituitary–gonadal (BPG) axis. Transcripts are clustered by average FPKM value. A total of 36 transcripts (28 complete and 8 partial ORFs) formed eight clusters (G1 to G8). The partial ORFs are indicated with their direction in parentheses. Red and blue indicate higher and lower expression, respectively. GenBank accession numbers of contigs used for clustering analysis are shown in Additional file 7: Table S6

In the RNA-seq analysis, three forms of Gnrh (herring-type Gnrh [*gnrh1*], chicken-II-type Gnrh [*gnrh2*], and salmon-type Gnrh [*gnrh3*]) and Gth subunits (*fshb*, *lhb*, and *cga*) were identified with their receptors, such as Gnrh receptors (*gnrhrs*), Fsh receptor (*fshr*), and Lh receptor (*lhr*). Of the *gnrhrs* identified, the deduced amino acid sequences of some transcripts showed high homology to three types of Gnrhr identified in medaka, that is, Gnrhr1, Gnrhr2, and Gnrhr3 (GenBank accession nos. NP_001098352, NP_001098392, and NP_001098393, respectively). The sequences of genes encoding steroidogenic proteins involved in ovarian steroidogenesis were also identified for *cyp11a1*, *cyp17a1*, *cyp19a1a*, *hsd3b*, and *star*. Additionally, eight types of 17β-hydroxysteroid dehydrogenase (*hsd17bs*; *hsd17b3*, *hsd17b4*, *hsd17b7*, *hsd17b8*, *hsd17b10*, *hsd17b12a*, *hsd17b12b*, and *hsd17b14*) were found. In addition to the above 24 genes, genes involved in the system of kisspeptin (Kiss)/Kiss receptor (*kiss1*, *kiss2*, and *kiss1r*) and thyroid-stimulating hormone (Tsh)/Tsh receptor (*tshb* and *tshr*), and genes of the other cytochromes P450 (*cyp1a1*, *cyp1b1*, and *cyp19a1b*) were selected to generate clustering of the differentially expressed reproduction-related genes in the BPG axis. For four receptor genes (*fshr*, *lhr*, *gnrhr1*, and *gnrhr3*), the 5′ and 3′ partial ORFs were found from the identified transcripts, while for other genes, the complete ORFs were found. A total of 36 transcripts (28 complete and 8 partial ORFs) were chosen to profile the abundance of reproduction-related transcripts in each tissue and they formed eight clusters (G1 to G8; Fig. 2). In the G1 cluster, the highest expression of *fshb*, *lhb*, and *cga* was observed in the pituitary. In the G2 cluster, six types of *hsd17bs* (*hsd17b4*, *hsd17b7*, *hsd17b8*, *hsd17b10*, *hsd17b12a*, and *hsd17b12b*) and *hsd3b* showed high expression in all tissues. In the G3 cluster, *tshb*, *tshr*, and *cyp1b1* showed high expression in the pituitary, whereas their expression was low in the gonad. In the G4 cluster, *fshr*, *lhr*, *cyp11a1*, *cyp17a1*, *hsd17b14*, and *star* were expressed mainly in the gonad. In the G5 and G6 clusters, *kiss1r*, *gnrh1*, *gnrh2*, *gnrhr2*, and *gnrhr3* were expressed mainly in the brain and pituitary. In the G7 cluster, *cyp1a1* and *cyp19a1a* were expressed mainly in the brain and gonad, and high expression of *cyp1a1* was observed in the ovary. In the G8 cluster, *kiss1*, *kiss2*, *gnrh3*, *gnrhr1*, and *cyp19a1b* were mainly expressed in the brain.

**Fig. 3:**
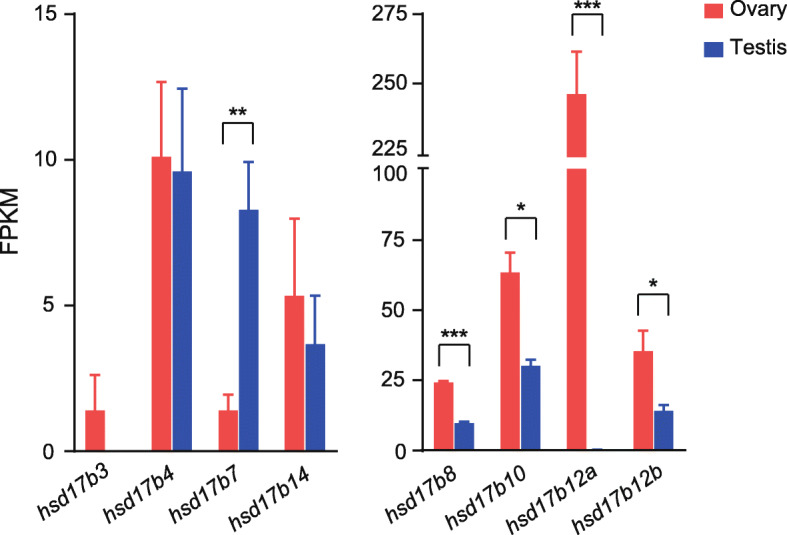
Comparison of transcription levels of 17β-hydroxysteroid dehydrogenases (*hsd17bs*) between ovary and testis. Asterisks indicate significant differences (*, *P* < 0.05; **, *P* < 0.01; ***, *P* < 0.001)

The transcription levels of *hsd17bs* were further compared between ovary and testis (Fig. 3). The expression of *hsd17b7* was higher in testis than in ovary. In contrast, the expression of *hsd17b8*, *hsd17b10*, *hsd17b12a*, and *hsd17b12b* was higher in ovary than in testis. *hsd17b3*, *hsd17b4*, and *hsd17b14* showed no difference in expression between ovary and gonad.

### Sequence alignments of ovarian steroidogenesis-related genes

Genes that play roles in the ovarian steroidogenic pathway were identified based on the KEGG database. Of these, cDNAs encoding Gth subunits (*fshb*, *lhb*, and *cga*), Gthrs (*fshr* and *lhr*), four steroidogenic enzymes (*cyp11a1*, *cyp17a1*, *cyp19a1a*, and *hsd3b*), and Star (*star*) were cloned and sequenced using a PCR-based strategy (Table 4; Additional file 4).

**Table 4:** Sequence information of Japanese sardine genes revealed by cDNA cloning

Gene symbol	Gene accession number	Nucleotide sequences of ORF (bp)	Amino acid sequences	Amino acid sequence similarity (%)		
				Atlantic herring	Zebrafish	Medaka
*fshb*	LC545605	387	128	57.3	48.9	38.3
*lhb*	LC545604	426	141	79.9	68.3	57.8
*cga*	LC545603	345	114	80.7	74.8	62.8
*fshr*	LC545601	2025	674	81.8	76.8	68.2
*lhr*	LC545602	2040	679	76.1	73.3	61.8
*cyp11a1*	LC545596	1560	519	94.0	82.1	82.4
*cyp17a1*	LC545597	1536	511	94.3	87.5	82.8
*cyp19a1a*	LC545598	1578	525	91.8	82.9	78.8
*hsd3b*	LC545599	1125	374	91.7	86.6	81.0
*star*	LC545600	864	287	94.4	88.2	89.5

Sequence alignments of the amino acid sequences and GenBank accession numbers of Atlantic herring (*Clupea harengus*), zebrafish (*Danio rerio*), and medaka (*Oryzias latipes*) are shown in Additional file 4

Multiple clones exhibiting different amino acid sequences for Fshb were obtained from ten pituitaries, where some showed a shift of an N-linked glycosylation site (see detailed explanation in Additional file 5). For real-time PCR, gene-specific primers for *fshb* were designed to include nucleotide sequences conserved in all cDNA sequences determined. In contrast, the deduced amino acid sequences of Lhb and Cga were highly conserved among different individuals. The deduced amino acid sequences of Japanese sardine Fshb, Lhb, and Cga are 57.3, 79.9, and 80.7% similarity to those of Atlantic herring, respectively. The Japanese sardine Fshb possesses 48.9 and 38.3% sequence similarity with zebrafish and medaka Fshb, but Lhb and Cga share 58–75% sequence similarity between them (Table 4; Additional file 4: Figure S2).

The deduced amino acid sequences of both Japanese sardine Fshr and Lhr are 81.8 and 76.1% similarity to those of Atlantic herring, respectively, while they possess 62–77% sequence similarities to those of zebrafish and medaka (Table 4; Additional file 4: Figure S3 and S4).

The deduced amino acid sequences of Japanese sardine steroidogenic proteins encoded by *cyp11a1*, *cyp17a1*, *cyp19a1a*, *hsd3b*, and *star* share high sequence similarities with those of Atlantic herring (92–94%), while they possess 79–90% sequence similarities to those of zebrafish and medaka (Table 4; Additional file 4: Figure S5–S9).

### Changes in ovarian development

To analyze the expression profiles of ovarian steroidogenesis-related genes using real-time PCR, female Japanese sardines at age 2 were sampled in September, October, December, and January, and fish at age 3 were sampled in September (Table 5). The body size was 157.0 ± 3.3 mm in BL and 41.7 ± 3.2 g in BW in September at age 2, whereas it was 184.5 ± 3.9 mm in BL and 78.8 ± 3.4 g in BW in September at age 3. The gonadosomatic index (GSI) was 0.44 ± 0.03 in September at age 2, and it increased to 2.63 ± 0.36 in January, but decreased to 0.48 ± 0.03 in September at age 3.

**Table 5:** Number of fish sampled, body length (BL), body weight (BW), gonadosomatic index (GSI), and ovarian developmental stage of female Japanese sardine sampled for quantitative PCR

Age	Sampling month	Number of fish	BL (mm)	BW (g)	GSI	Ovarian development
2	September	5	157.0 ± 3.3	41.7 ± 3.2	0.44 ± 0.03	Immature
	October	6	167.3 ± 4.7	57.1 ± 7.1	1.02 ± 0.08	Previtellogenic growth
	December	5	173.4 ± 4.4	70.5 ± 7.4	1.51 ± 0.43	Mid-vitellogenesis
	January	5	184.6 ± 3.4	79.1 ± 4.3	2.63 ± 0.36	Late vitellogenesis
3	September	4	184.5 ± 3.9	78.8 ± 3.4	0.48 ± 0.03	Immature

**Fig. 4:**
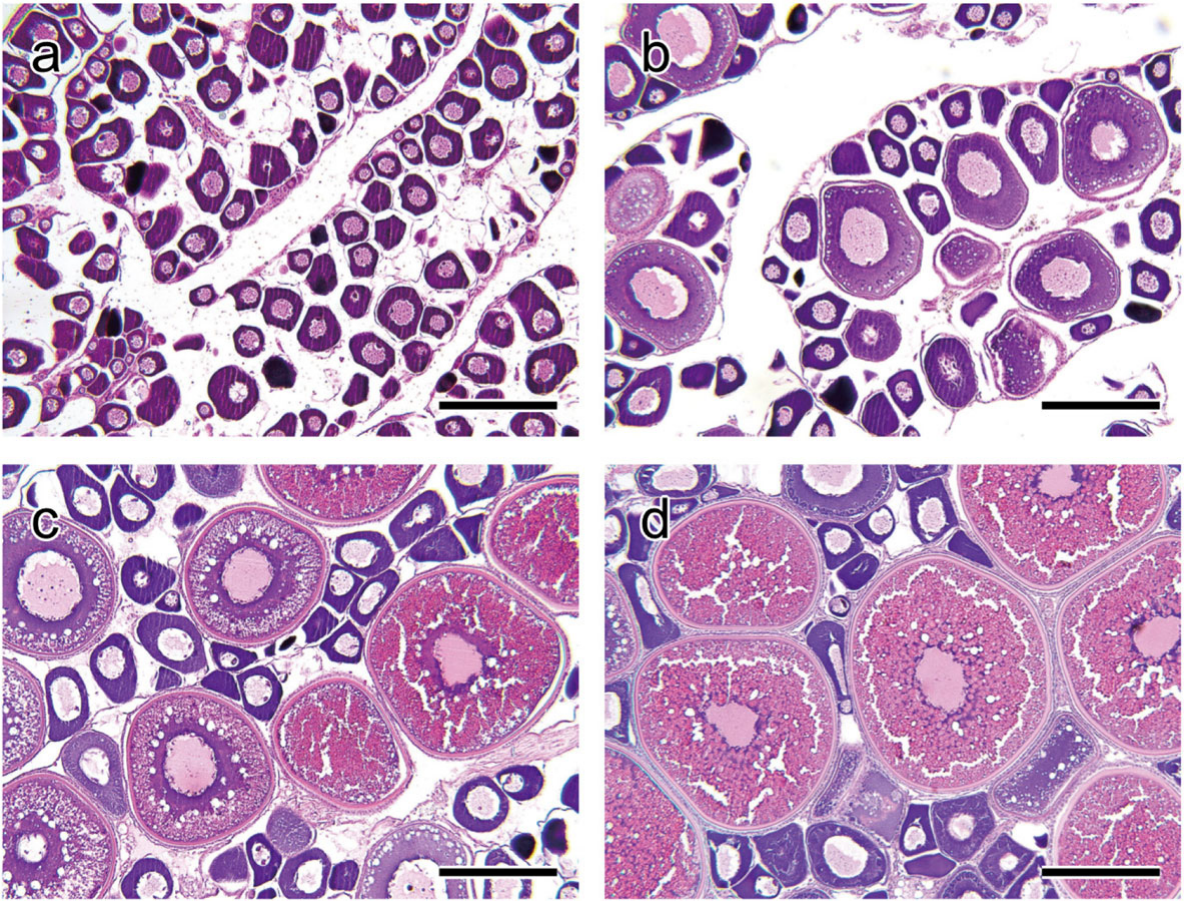
Histological micrographs of Japanese sardine ovaries at various stage of development. Ovarian developmental stages are as follows: immature, **a**; previtellogenic growth, **b**; mid-vitellogenesis, **c**; late vitellogenesis, **d**. Bars = 200 μm

In September, all fish at ages 2 and 3 had immature ovaries, occupied by perinucleolus-stage oocytes (Fig. 4a). In October, most fish had ovaries containing cortical alveolus-stage oocytes, indicating the progress of previtellogenic growth (Fig. 4b). In December, some females were at the middle phase of vitellogenesis (Fig. 4c), while in January, most females were at the late phase of vitellogenesis (Fig. 4d).

### Changes in ovarian steroidogenesis-related gene expressions

**Fig. 5:**
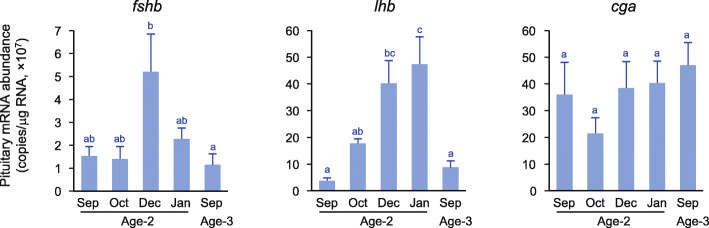
Changes in pituitary gene expressions of gonadotropin (Gth) subunits (*fshb*, *lhb*, and *cga*). Different letters indicate significant differences (*P* < 0.05)

The expression of *fshb* was highest in December at age 2, while it was lowest in September at age 3 (Fig. 5). The expression of *lhb* was lowest in September at age 2, gradually increased, peaked in January and declined in September at age 3. The expression of *cga* did not change significantly throughout the experimental period.

**Fig. 6:**
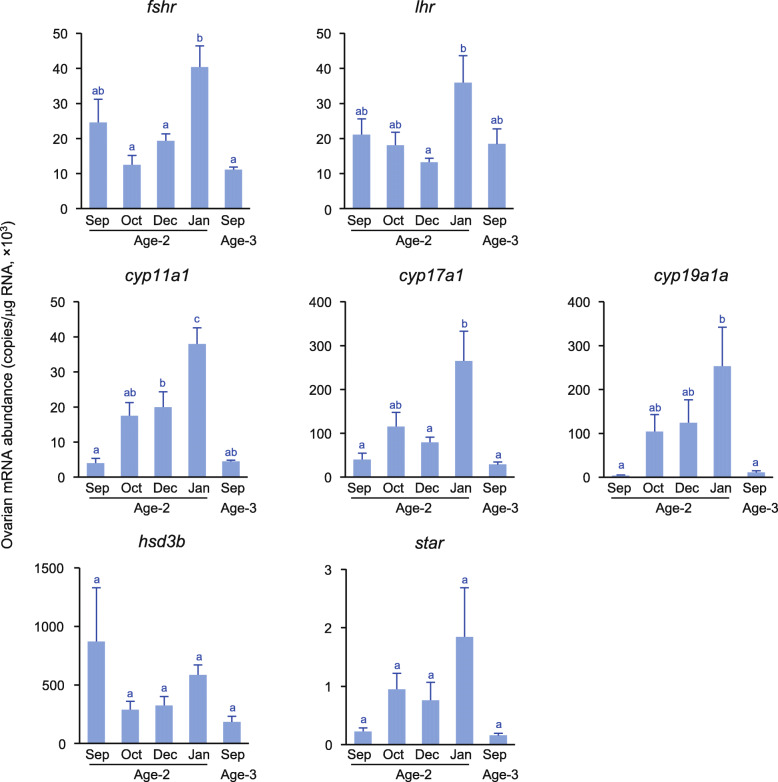
Changes in ovarian gene expressions of Gth receptors (Gthrs; *fshr* and *lhr*), steroidogenic enzymes (*cyp11a1*, *cyp17a1*, *cyp19a1a*, and *hsd3b*), and steroidogenic acute regulatory protein (*star*). Different letters indicate significant differences (*P* < 0.05)

The expression of *fshr* was lowest between October and December at age 2, increased in January, but decreased in September at age 3 (Fig. 6). A significant increase in *lhr* expression was found from December to January at age 2. The expression of *cyp11a1*, *cyp17a1*, and *cyp19a1a* was low in September at age 2 and then increased, peaked in January but decreased in September at age 3. The expression of *hsd3b* and *star* did not change significantly throughout the experimental period, although the level of *star* tended to be higher in January.

### Changes in serum steroid hormone levels

**Fig. 7:**
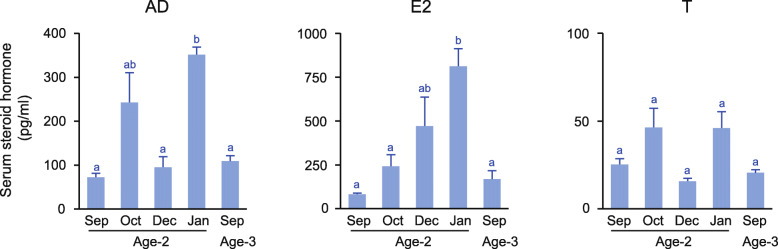
Changes in serum levels of androstenedione (AD), estradiol-17β (E2), and testosterone (T). Different letters indicate significant differences (*P* < 0.05)

Serum androstenedione (AD) levels were low in September and December at ages 2 and 3 (72.2 ± 9.5 to 109.3 ± 11.9 pg/ml), while they were the highest in January at age 2 (351.4 ± 17.2 pg/ml) (Fig. 7). Serum E2 levels were low in September at age 2 (82.2 ± 7.4 pg/ml), gradually increased from October, peaked in January (812.1 ± 100.4 pg/ml), and then declined in September at age 3 (169.3 ± 48.2 pg/ml). Serum testosterone (T) levels remained low throughout the experimental period (below 46.2 ± 11.1 pg/ml). Serum estrone (E1) levels were below the assay detection limit (< 15 pg/ml) in the serum of all fish.

## Discussion

### Japanese sardine transcriptome in the BPG axis

In the present study, a total of 115,173 ORFs were predicted from transcripts in the BPG axis of Japanese sardine. Of these, about 40% of protein sequences have similarity to Atlantic herring, zebrafish, and medaka (Fig. 1a). In addition, the distribution of percent identity of amino acids indicated that the sardine protein sequence is more identical to Atlantic herring than to zebrafish and medaka (Fig. 1b). In the genomes of Atlantic herring, zebrafish, and medaka, 43,000–53,000 proteins have been annotated [13, 26–28]. In the present study, the use of these three species as a reference detected about half of the proteins, for example, 24,037 proteins of Atlantic herring out of 47,201 proteins of the sardine (Fig. 1a). Among those proteins, we succeeded in obtaining sequence information of many key genes involved in the BPG axis of Japanese sardine.

A Venn diagram indicated that the numbers of tissue-specific transcripts in the brain and pituitary were similar between female and male, while in the gonad this number was higher in testis than in ovary (Fig. 1c). Similar trends for genes differentially expressed between ovary and testis have been reported in some fish species, such as bluehead wrasse (*Thalassoma bifasciatum*) [29], Nile tilapia (*Oreochromis niloticus*) [30], and sharpsnout seabream (*Diplodus puntazzo*) [31], where many more genes were expressed in testis than in ovary. Male-biased gene expression may be a general characteristic feature of fish gonad. Additionally, in the wrasse, such sex-biased gene expression in gonad was more potent than that in brain [29], similar to the tissue-specific expression patterns observed in the sardine.

The genes differentially expressed between ovary and testis were further characterized by the enriched GO terms. The majority of top GO terms enriched in the ovary were related to oogenesis and sperm–egg recognition, while those in the testis were related to spermatogenesis and cilium function, which may be linked to formation of the sperm flagellum. Thus, many genes differentially expressed between ovary and testis were directly linked to gamete production in each gonad. These results will provide us with numerous candidate genes potentially responsible for regulating gametogenesis in future study of the sardine.

### Differential expression of reproduction-related genes in the BPG axis

Many genes encoding the major regulators in the BPG axis were identified in the Japanese sardine, and their transcription levels showed tissue- and sex-specific expression patterns (Fig. 2).

As in other vertebrates, teleost Gths are synthesized in gonadotropic cells in the pituitary, in which transcripts of Gth subunits are abundant [14]. Indeed, the highest expression of *fshb*, *lhb*, and *cga* was detected in the sardine pituitary. We also found high expression of *tshb* in the sardine pituitary. Tsh is composed of common Cga and hormone-specific β-subunit (Tshb) and produced in the pituitary like Gth, but it exhibits various physiological functions, such as in growth and metamorphosis including reproduction [32]. Teleost Tshr has been detected in various tissues, such as gonad, brain, kidney, and gills [33, 34]. In the present study, high expression of sardine *tshr* was found in the pituitary.

As in other teleosts, transcripts encoding three forms of Gnrh (*gnrh1*, *gnrh2*, and *gnrh3*) were identified within the present annotated sequences, where Gnrh1 is the herring type that is found in fish belonging to the order Clupeiformes [35]. In fish brain, different expression of the three *gnrhs* has been found in a variety of regions, while Gnrh1 is suggested to be the main regulator of Gth secretion because Gnrh1-expressing neurons innervate into the pituitary [16, 36]. In the present study, *gnrh1* and *gnrh2* were mainly expressed in the brain and pituitary, while *gnrh3* was expressed mainly in the brain. Numerous Gnrhrs have been identified in teleost, but their classification has not been fully established [37]. In medaka, three types of *gnrhr* (*gnrhr1*, *gnrhr2*, and *gnrhr3*) have been characterized in detail [38, 39]. We also found these three types in the Japanese sardine, showing that the *gnrhr2* and *gnrhr3* expression was mainly detected in the brain and pituitary, whereas *gnrhr1* was in the brain. This suggests that, in the sardine, Gnrhr2 and Gnrhr3 are plausible candidates for mediating signal transduction of Gnrh-stimulated Gth response. While the Kiss/Kissr system has been proposed to be involved in the neuroendocrine control of the BPG axis in fish as recognized in mammals, the physiological roles in fish reproduction are still not well defined [40–42]. In the present study, the expression of *kiss1* and *kiss2* was mainly detected in the sardine brain.

Five genes (*fshr*, *lhr*, *cyp11a1*, *cyp17a1*, and *star*) expressed mainly in the ovary are well known to play major roles in the ovarian steroidogenic pathway [19]. Aromatase also plays a central role in the biosynthesis of estrogen, and teleosts possess two types of aromatase gene: ovarian-type *cyp19a1a* and brain-type *cyp19a1b* [43]. In the present study, *cyp19a1a* was expressed in the brain and gonad, whereas *cyp19a1b* was mainly expressed in the brain, suggesting that the sardine ovarian aromatase is encoded by *cyp19a1a*, as in other fishes. Hsd3b is another critical enzyme in ovarian steroidogenesis. In the present study, high expression of *hsd3b* was found in the gonad, while it was also expressed in the brain and pituitary. A study in stinging catfish (*Heteropneustes fossilis*) showed that *hsd3b* is widely expressed in the brain, suggesting its role in neurosteroidogenesis [44]. Cytochrome P450 1A1 (*cyp1a1*) and 1B1 (*cyp1b1*) play roles in E2 metabolism in mammals, while *cyp1a1* is suggested to be primarily responsible for this function in zebrafish [45]. In the present study, *cyp1a1* expression was mainly found in the brain and gonad, especially in the ovary, whereas *cyp1b1* was expressed mainly in the brain and pituitary, suggesting a specific role of *cyp1a1* in the ovary like in zebrafish. Finally, we also found high expression of six types of *hsd17b* (*hsd17b4*, *hsd17b7*, *hsd17b8*, *hsd17b10*, *hsd17b12a*, and *hsd17b12b*) in all three tissues. We further compared the transcription levels of *hsd17bs*, focusing on their sex-specific expression.

Hsd17b catalyzes the interconversion of 17-ketosteroids to 17β-hydroxysteroids, such as E1 to E2, and a total of 15 Hsd17b family genes have been identified in mammals [46, 47]. In fish, several different *hsd17b* transcripts have been isolated, showing differential tissue-specific expression and catalytic activities with various types of Hsd17b [48–50]. However, the specific function of each Hsd17b in ovarian steroidogenesis has not been clearly confirmed. The present study identified eight types of Hsd17b in the Japanese sardine. By comparing transcription levels between ovary and testis, higher expression of *hsd17b8*, *hsd1710*, *hsd17b12a*, and *hsd17b12b* was found in the ovary, whereas higher expression of *hsd17b7* was found in the testis (Fig. 3). A study of the Japanese eel (*Anguilla japonica*) revealed that Hsd17b12a is one of the enzymes mediating the biosynthesis of 11-ketotestosterone, which is the main androgen in fish spermatogenesis [49]. Therefore, it is interesting that *hsd17b12a* was found to be most highly expressed in the sardine ovary. In mammals, Hsd17b1 expressed in granulosa cells play a central role in estrogen biosynthesis, and Hsd17b7 also catalyzes the conversion of E1 to E2 in the ovary and placenta [51]. However, the sequence of the *hsd17b1* was not obtained in our data, and *hsd17b7* was found but showed higher expression in testes than in ovary. Accordingly, while our results suggest sex-specific differences in the function of each Hsd17b in the sardine, it is still difficult to suggest which type would have a role in estrogen biosynthesis in this species. The present sequence data will allow future studies on the physiological roles of Hsd17bs in sardine reproduction, which should be useful for understanding the gonadal steroidogenic pathway unique to teleost fish.

### Molecular cloning of ovarian steroidogenesis-related genes

A total of 10 genes that have critical roles in ovarian steroidogenesis were selected, and the cDNAs were cloned and sequenced. The deduced amino acid sequences of Japanese sardine Cga and Lhb share approximately 80% sequence similarity with their homologs in Atlantic herring, whereas Fshb shares only 57% similarity. The sequences and structures of Cga and Lhb are generally more conserved than those of Fshb in teleosts [14]. Likewise, the sequences of Lhr are more conserved than those of Fshr among vertebrate species [14]. However, the sequence similarities of sardine Lhr to those in zebrafish and medaka were lower than those of Fshr. In contrast, the sequences of steroidogenic proteins are highly conserved among teleosts [52]. In accordance with this, we found high sequence similarities (> 80%) of steroidogenic proteins encoded by *cyp11a1*, *cyp17a1*, *cyp19a1a*, *hsd3b*, and *star* between sardine and other fish species.

For sardine Fshb, multiple *fshb* clones were isolated from transcripts of ten pituitaries (Additional file 5). This suggests that the sardine Fshb may have many amino acid sequence variants between individuals. In contrast, the existence of isoforms of Gth subunits has been reported in some fish species such as zebrafish and salmonids and this seems to be derived from tetraploidy of the fish species [53]. Genomic analysis of Atlantic salmon (*Salmo salar*) revealed that its genome exhibits largely tetraploid due to a genome duplication, showing a high repeat content (58–60%) [54]. Therefore, it is also important to consider the possibility of the existence of Fshb paralogues in the sardine. Another sequencing approach targeting the isoforms and variants might be required to confirm this association.

### Expression profiles of ovarian steroidogenesis-related genes

**Fig. 8:**
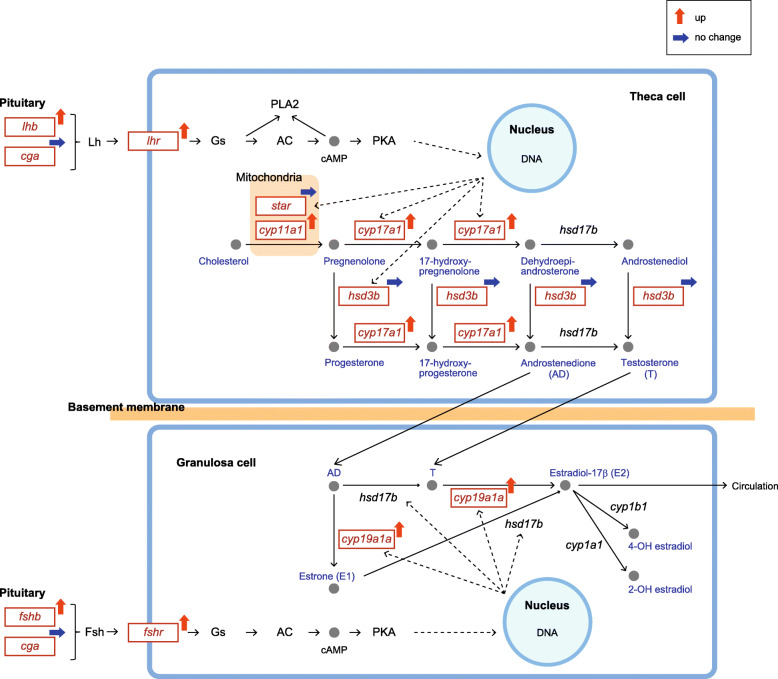
Diagram of ovarian steroidogenic pathway adapted from KEGG. Genes related to the Gth/Gthr system and steroid hormone biosynthesis are indicated. Genes identified by molecular cloning in the Japanese sardine are shown in red boxes. Their expression profiles were confirmed by quantitative PCR. Genes that were upregulated or whose expression did not change during ovarian development (vitellogenesis) are marked with red and blue arrows, respectively (top right of gene names). The localization of each gene and the steroidogenic pathways in ovarian follicles are still not clarified in the sardine

The present study and other previous studies [55, 56] showed that serum E2 levels in the Japanese sardine gradually increased during ovarian development. This was accompanied by increased serum vitellogenin levels [55]. In the present study, we focused on the ovarian steroidogenic pathway and analyzed the expression profiles of ovarian steroidogenesis-related genes in relation to the vitellogenic process (Fig. 8).

The physiological role of Fsh has been studied in detail in salmonids, showing that Fsh stimulates the production of E2 in follicle cells and induces vitellogenin uptake [15, 57, 58]. Conversely, the major physiological role of Lh is well recognized in many teleost species as a main regulator of the production of maturation-inducing steroid, which initiates the final phase of oocyte development, that is oocyte maturation and ovulation [59]. In the present study, the peak of pituitary *fshb* expression was found during the middle phase of vitellogenesis, whereas *lhb* expression peaked at the completion of vitellogenesis. This difference may reflect a shift of the activation of Gth synthesis from Fsh to Lh in association with a shift from a vitellogenic process to oocyte maturation. Pituitary *cga* expression did not change throughout ovarian development, suggesting that *cga* expression is kept constant regardless of the activation of Gth synthesis.

The present study demonstrated that, in the sardine ovary, the expression of *fshr* and *lhr* was upregulated during the late phase of vitellogenesis. The size-dependent separation of ovarian follicles (oocytes surrounded by follicle cell layers) revealed that *fshr* expression was elevated during vitellogenesis, peaking at the completion of vitellogenesis, and then decreased rapidly after the initiation of oocyte maturation [60]. In contrast, *lhr* expression is upregulated at the completion of vitellogenesis and maintained at a high level during the early phase of oocyte maturation. Thus, in individual ovarian follicles, elevated *fshr* expression during vitellogenesis is switched to rapid expression of *lhr* by the completion of vitellogenesis, preparing for the initiation of oocyte maturation. Although we did not analyze the oocyte size-dependent gene expression, the significant increases in *fshr* and *lhr* expression in the ovary at the completion of vitellogenesis may reflect the active egg production in preparation for spawning.

In salmonids, the ovarian gene expression of steroidogenic proteins (*cyp11a1*, *cyp17a1*, *cyp19a1a*, *hsd3b*, and *star*) is upregulated during vitellogenesis, in correlation with the production of E2 [61, 62]. In the present study, the expression of *cyp11a1*, *cyp17a1*, and *cyp19a1a* was lowest in immature ovary, gradually increased during ovarian development, and peaked at the completion of vitellogenesis. However, no significant changes in the expression of *star* and *hsd3b* occur, although *star* expression tended to be higher in the late-vitellogenic ovary than in immature ovary. In salmonids, Fsh upregulated *cyp11a1*, *cyp17a1*, and *cyp19a1a* expression in the previtellogenic ovarian follicles, but it was more potent in inducing *star* and *hsd3b* expression [63, 64]. Such differences in the Fsh response to gene expression may lead to differential expression profiles found in sardine steroidogenic genes.

The in vitro cultivation of ovarian follicles with radiolabeled steroid precursors has revealed that E2 is synthesized via T in ovarian follicles of chub mackerel (*Scomber japonicus*) and medaka, whereas E2 is synthesized via E1 in bambooleaf wrasse (*Pseudolabrus japonicus*) and red seabream (*Pagrus major*) [65–68]. Thus, there are species differences in the main substrate precursor of E2. In the present study, the serum levels of AD, which is the substrate precursor of T and E1, were elevated during ovarian development. Since aromatase catalyzes the conversion of T to E2 and AD to E1, the upregulation of *cyp19a1a* during ovarian development suggests that the catalytic activity of aromatase on E2 conversion from T or E1 conversion from AD may be activated. However, serum levels of T remained low throughout ovarian development, consistent with previous reports on sardine [55, 56]. We also found that E1 was not detected in the serum of female sardines. Hence, there is a need for further research on the E2 synthetic pathway of sardine ovary. Here, the physiological characterization of each type of Hsd17 will be important to consider since Hsd17b catalyzes the activation of AD to T and E1 to E2.

## Conclusions

In the present work, we have identified reproduction-related genes from the BPG axis of Japanese sardine using NGS techniques. Genes encoding major regulators in the BPG axis were characterized in terms of their tissue- and sex-specific expression. Here, four of eight Hsd17b genes (*hsd17b8*, *hsd17b10*, *hsd17b12a*, and *hsd17b12b*) identified showed higher expression in ovary than in testis. This is of particular interest since the function of Hsd17bs in ovarian steroidogenesis is not well established. Despite the ecological and commercial importance worldwide, transcriptome information has rarely been available in the physiological study of clupeoid fish. The present transcriptomic sequences will provide a valuable resource for studying the molecular biology of clupeoid fish.

In the present study, a detailed molecular picture of the ovarian steroidogenic pathway was indicated from our transcript data of endocrine factors (Fig. 8). The information provided a good foundation for understanding the molecular mechanisms in the physiological process of sardine ovarian development. External factors such as temperature and feeding, and internal factors such as age and growth are known to influence gene expression in the BPG axis [14, 69]. Additional studies on the particular endocrine factors and the responses to changes in external and internal factors should provide further insight into the endocrine regulation of ovarian development in this species. Studies on wild sardine have demonstrated that characteristics of ovarian development such as fecundity and timing of maturity varied in association with changes in population size [23, 24]. Therefore, the physiological regulation of ovarian development at an individual level may lead to a new understanding of mechanisms associated with population fluctuation in the sardine.

Lastly, RNA-seq analysis of the reproduction-related transcriptome can serve as a foundation for studying the reproductive physiology of fisheries’ target species. We performed captive experiments since these often provide useful information for understanding the reproductive characteristics of fish with high reproducibility [70]. Although specific traits in wild fish as compared to captive fish should also be considered, endocrine regulation of gonadal development, as well as molecular properties, might be conserved within each species. A combination of molecular analysis and field-research-dependent biological data would open up interesting avenues for the study of reproductive biology and stock assessment.

## Methods

### Fish and sample collection

The Japanese sardine used in this study were commercially caught in the wild as juveniles (0+ years old). About 400 fish were transferred to indoor concrete tanks at Hakatajima Field Station, Fisheries Technology Institute (formerly Hakatajima Laboratory, National Research Institute of Fisheries and Environment of Inland Sea), Japan Fisheries Research and Education Agency, Kinoura, Imabari, Ehime, Japan. The fish were then maintained for 1 to 2 years. Throughout the maintenance of the general farming system, the fish were regularly fed with commercial dry pellets. Of those reared, a total of 31 fish was used in the present study. On 18 February 2017, three females and three males were sampled at age 3. This period was chosen since the expression of many genes related to the BPG axis is activated during active gonadal development [14, 61]. For each fish, brain, pituitary, and a portion of gonad were frozen and stored at − 80 °C for RNA-seq analysis. Other fish were sampled on 17 October and 4 December, 2017, and 16 January and 18 September, 2018. Five to six females at age 2 were taken in October, December, January, and September, while four females at age 3 were taken in September. The BL, BW, and gonadal weight (GW) of each specimen were measured. Blood samples were centrifuged (3500×g, 15 min, 4 °C), and the serum was collected and stored at − 30 °C for steroid hormone measurements. A portion of each ovary was fixed in Bouin’s solution for histological observation. The rest of the ovary and pituitary were immersed in RNAlater (Qiagen, Hilden, Germany) for 24 h and then stored at − 80 °C for molecular cloning and quantitative real-time PCR analysis. The GSI was calculated as follows: GSI = (GW / BW) × 100. In total, 25 fish were used for the quantitative analysis of gene expression and steroid hormones (Table 5). The sample size of *n* = 4–6 per group was determined according to previous studies on fish [61, 71]. For each sampling, the fish were euthanized by rapid decapitation. The treatment of fish adhered to the guidelines for animal experiments set by the National Research Institute of Fisheries and Environment of Inland Sea.

### Gonadal histology

A portion of each gonad fixed in Bouin’s solution was dehydrated through a graded series of ethanol. Dehydrated tissues were embedded in paraffin. Sections were cut (5 μm) and stained with hematoxylin–eosin.

### RNA extraction and sequencing

Total RNA was extracted from brain, pituitary, and gonad (three fish for each sex) that had been stored at − 80 °C using a TRIzol Plus RNA Purification Kit (Thermo Fisher Scientific, Waltham, MA, USA) with PureLink DNase (Thermo Fisher Scientific), in accordance with the manufacturer’s instructions. The concentration and quality of purified total RNA were determined using a Qubit RNA HS Assay Kit (Thermo Fisher Scientific) and a Bioanalyzer 2100 with RNA 6000 Nano Kit (Agilent Technologies, Inc., Santa Clara, CA, USA).

The cDNA libraries were constructed using the total RNA (1 μg) and SuperScript III reverse transcriptase (Thermo Fisher Scientific), in accordance with the TruSeq RNA Sample Prep ver. 2 (LS) protocol (Illumina, Inc., San Diego, CA, USA). The cDNA libraries were sequenced into 150-bp paired-end reads by NextSeq500 (Illumina, Inc.) at the Bioinformatics and Biosciences Division of the Fisheries Resources Institute (formerly Research Center for Bioinformatics and Biosciences of the National Research Institute of Fisheries Science), Yokohama, Japan.

### Transcriptome assembly and annotation

The assembly was performed as previously described [72]. Briefly, the sequence trimming was performed using Trimmomatic [73], and the quality confirmation was performed using FastQC [74]. The remaining paired-end reads were assembled using Trinity [75]. From the assembled sequences, ORFs of more than 300 bp were extracted and translated into protein sequences using TransDecoder [76]. ORFs of 95% similar protein sequences were clustered using the CD-HIT program [77] to remove redundant protein sequences.

The proteins were annotated based on their homology to protein sequences of the Atlantic herring, zebrafish, and medaka, using the BLASTP program, with a threshold e-value of <1e–05. A homology search was also performed using DIAMOND software [78] and the NCBI nr database with a threshold e-value of <1e–05. The cumulative relative frequency for amino acid identity against each database was plotted using R software ver. 3.3.1 [79]. The GO and EC numbers, which are shared with the accession numbers used in zebrafish and medaka, were assigned from the best hits of the BLASTP results against these two species. The retrieved accession numbers of both zebrafish and medaka were converted to UniProt accession numbers at the UniProt website (https://www.uniprot.org/) and then GO terms were retrieved from the UniProt sequence data. The retrieved GO terms were converted to GO-slim which is cut-down version of GO [80] by OmicsBox software ([81]; https://www.biobam.com/omicsbox). Based on the assigned EC numbers and annotated descriptions in Japanese sardine, the protein sequences related to reproduction were obtained referring to the Gnrh signaling pathway (Pathway ID: 04912) and steroid hormone biosynthesis (Pathway ID: 00140) in Kyoto Encyclopedia of Genes and Genomes (KEGG).

### Differential expression analysis

The transcription level of each gene was determined from the number of mapped reads. The mapping of reads to each transcript and the counting were performed using Bowtie2 [82] and RSEM [83], respectively. Transcription levels were normalized among the libraries using the trimmed mean of M-values method [84] and statistically compared with the edgeR package [85] in R software ver. 3.3.1. Fisher’s exact test was performed in OmicsBox software to estimate whether the differentially expressed genes were particularly associated with specific GO categories when compared with the background genes. *P*-values were adjusted by FDR [86], and the FDR of < 0.05 was chosen as the threshold for significance. The normalized fragments per kilobase of transcript per million mapped read (FPKM) of genes was used for generating Venn diagrams and a heatmap with R software ver. 3.3.1.

### cDNA cloning

The complete nucleotide sequences for cDNAs encoding Japanese sardine Gth subunits (*fshb*, *lhb*, and *cga*), Gthrs (*fshr* and *lhr*), three cytochrome P450 enzymes (*cyp11a1*, *cyp17a1*, *cyp19a1a*), Hsd3b (*hsd3b*), and Star (*star*) were determined using basic molecular cloning techniques as previously described [71]. *fshb*, *lhb*, and *cga* were cloned from the pituitary, while *fshr*, *lhr*, *cyp11a1*, *cyp17a1*, *cyp19a1a*, *hsd3b*, and *star* were cloned from the ovary. Total RNA was extracted from the pituitary using an RNeasy Lipid Tissue Mini Kit (Qiagen), while total RNA was extracted from the ovary using an RNeasy Mini Kit (Qiagen). RNA extracts were treated with DNase using the Turbo DNA-free Kit (Thermo Fisher Scientific). First-strand cDNA was synthesized using a Superscript III reverse transcriptase (Invitrogen, Carlsbad, CA, USA) with random hexamer primers. For *fshb*, *lhb*, and *cga*, the cDNAs from four pituitaries were subjected to PCR amplification. Since several transcripts encoding different amino acid sequences of Fshb were isolated, the cDNAs from the other six pituitaries were further used for cloning of *fshb*. For other genes, the cDNAs from two individuals were subjected to PCR amplification.

Based on the sequences of contigs, primers were designed in the 5′ and 3′ untranslated region (5′ and 3′ UTR) to obtain the clones containing full ORFs (Additional file 6: Table S3). For *fshb*, *lhb*, *cga*, *cyp11a1*, *cyp17a1*, *cyp19a1a*, *hsd3b*, and *star*, these contigs contain the complete ORF with the 5′ and 3′ UTR. For *fshr* and *lhr*, only contigs containing 5′ and 3′ ORFs were found. GenBank accession numbers of the contigs used for designing primers are listed in Additional file 7: Table S6.

The similarity of protein-coding sequences was calculated through pairwise alignment using BioEdit software.

### Quantitative PCR

Expression levels of pituitary and ovarian genes were determined by real-time PCR using a Thermal Cycler Dice Real Time System (Takara, Shiga, Japan). Gene-specific primers were designed for *fshb*, *lhb*, *cga*, *fshr*, *lhr*, *cyp11a1*, *cyp17a1*, *cyp19a1a*, *hsd3b*, and *star* (Additional file 6: Table S4 and S5). Although several isoforms of Fshb were found in the molecular cloning, primers for real-time PCR were designed to count all *fshb* isoforms because it is currently unclear whether these isoforms exhibit different functions.

Using the same methods as described in the section on cDNA cloning, total RNAs were extracted from the pituitary and from a piece of ovary, and template cDNAs were synthesized after DNase treatments. For each PCR reaction, 20 μl of PCR reaction mix was prepared, which contained 10 μl of TB Green Premix Ex Taq II (Takara), 0.6 μM of forward and reverse primers, and template cDNA corresponding to 0.3 ng (pituitary) or 50 ng (ovary) of total RNA. Real-time PCR runs were performed in 96-well plates in duplicate with no template controls. The thermal cycling conditions were 30 s at 95 °C, and 40 cycles of 5 s at 95 °C and 30 s at 60 °C. For each PCR, a standard curve from serial dilutions of purified PCR fragments containing a partial sequence of a target gene was constructed for quantification.

### Steroid hormone measurements

Serum AD was measured using time-resolved fluoroimmunoassay (TR-FIA), as previously described [87]. Serum E1, E2, and T were measured using the enzyme-linked immunosorbent assay (ELISA) kit (Abnova, CA, USA for E1; Cayman Chemical, MI, USA for E2 and T), in accordance with the manufacturer’s instructions. Before measurements of AD, E2, and T, the serum steroids were extracted into diethyl ether, which was then evaporated, and the remaining steroids were dissolved in suitable assay buffers for each assay. In the TR-FIA, AD-3-carboxymethyloxime-bovine serum albumin (AD-3-CMO-BSA) and anti-AD-3-CMO-BSA were used as coating antigen and primary antibody, respectively. All samples were assayed in duplicate for both TR-FIA and ELISA.

### Statistical analysis

Data are presented as mean ± standard error of the mean (SEM). GraphPad Prism version 8.00 for Windows (GraphPad Software, La Jolla, CA, USA) was used for statistical evaluation. Multiple paired comparisons of FPKM values between ovary and testis were made using the Holm–Sidak method (α = 0.05). Results of gene expressions from real-time PCR were analyzed by one-way ANOVA followed by Tukey’s multiple comparisons test.

## Supplementary information

**Additional file 1 :** Results of homology search against Atlantic herring (*Clupea harengus*), zebrafish (*Danio rerio*), and medaka (*Oryzias latipes*).

**Additional file 2 : Figure S1.** GO classification of assembled contigs.

**Additional file 3 :** Complete list of enriched GO terms in the gonads. **Table S1.** Enriched GO terms in the ovary. **Table S2.** Enriched GO terms in the testis.

**Additional file 4 :** Sequence alignments of the amino acid sequences of Gth subunits (**Figure S2**), Gthrs (**Figure S3 and S4**), and steroidogenic proteins (**Figure S5–S9**).

**Additional file 5 :** Molecular characterization of Japanese sardine Fshb. **Figure S10.** Sequence alignment of the amino acid sequences of Japanese sardine Fshb isoforms.

**Additional file 6 :** Primers used for cDNA cloning and real-time PCR. **Table S3.** Primers used for cloning cDNAs. **Table S4.** Primers used for amplifying PCR products to construct a standard curve. **Table S5.** Primers used for real-time PCR.

**Additional file 7 : Table S6.** GenBank accession numbers of contigs used for the clustering analysis.

## Data Availability

The raw RNA sequencing reads from the Japanese sardine (*Sardinops melanostictus*) brain, pituitary, and gonad are available in DRA division of DDBJ under accession number DRA010129 (http://trace.ddbj.nig.ac.jp/DRASearch/submission?acc=DRA010129). The assembled contigs are also available in TSA division of DDBJ under accession numbers ICPT01000001–ICPT01290119 (http://getentry.ddbj.nig.ac.jp/top-e.html). The results of Protein Basic Local Alignment Search Tool are shown in Additional file 1. The GO ID linked to enriched GO terms are shown in Additional file 3. GenBank accession numbers for the sequences determined in this work are LC545596–LC545610. The reference genomes were obtained from the NCBI database (Atlantic herring [*Clupea harengus*], GCF_900700415.1; zebrafish [*Danio rerio*], GCF_000002035.6; medaka [*Oryzias latipes*], GCF_002234675.1). GenBank accession numbers used for sequence similarity calculation are included in Additional file 4. GenBank accession numbers of contigs used for clustering analysis are shown in Additional file 7.

## Abbreviations

AD: Androstenedione
BL: Body length
BLASTP: Protein Basic Local Alignment Search Tool
BPG axis: Brain–pituitary–gonadal axis
BSA: Bovine serum albumin
BW: Body weight
cDNA: Complementary DNA
CMO: Carboxymethyloxime
Cga: Glycoprotein hormones, alpha polypeptide
Cyp1a1: Cytochrome P450, family 1, subfamily A, polypeptide 1
Cyp1b1: Cytochrome P450, family 1, subfamily B, polypeptide 1
Cyp11a1: Cytochrome P450, family 11, subfamily A, polypeptide 1
Cyp17a1: Cytochrome P450, family 17, subfamily A, polypeptide 1
Cyp19a1a: Cytochrome P450, family 19, subfamily A, polypeptide 1a
Cyp19a1b: Cytochrome P450, family 19, subfamily A, polypeptide 1b
DDBJ: DNA Data Bank of Japan
DRA: DDBJ Sequence Read Archive
E1: Estrone
E2: Estradiol-17β
EC: Enzyme commission
ELISA: Enzyme-linked immunosorbent assay
FDR: False discovery rate
FPKM: Fragments per kilobase of transcript per million mapped read
Fsh: Follicle-stimulating hormone
Fshb: Follicle-stimulating hormone subunit beta
Fshr: Follicle-stimulating hormone receptor
Gnrh: Gonadotropin-releasing hormone
Gnrhr: Gonadotropin-releasing hormone receptor
GO: Gene Ontology
Gth: Gonadotropin
Gthr: Gonadotropin receptor
GSI: Gonadosomatic index
GW: Gonadal weight
Hsd17b: 17β-hydroxysteroid dehydrogenase
Hsd3b: 3β-hydroxysteroid dehydrogenase
KEGG: Kyoto Encyclopedia of Genes and Genomes
Kiss: Kisspeptin
Kissr: Kisspeptin receptor
Lh: Luteinizing hormone
Lhb: Luteinizing hormone subunit beta
Lhr: Luteinizing hormone receptor
NCBI: National Center for Biotechnology Information
NCBI nr database: NCBI non-redundant protein database
NGS: Next-generation sequencing
RNA-seq: RNA sequencing
ORF: Open reading frame
SEM: Standard error of the mean
Star: Steroidogenic acute regulatory protein
T: Testosterone
TR-FIA: Time-resolved fluoroimmunoassay
TSA: Transcriptome shotgun assembly
Tsh: Thyroid-stimulating hormone
Tshr: Thyroid-stimulating hormone receptor
UTR: Untranslated region
