# Supplementary material for: Transcriptome characterization of BPG axis and expression profiles of ovarian steroidogenesis-related genes in the Japanese sardine
Source: BMC Genomics. 2020 Sep 29;21:668. doi: 10.1186/s12864-020-07080-1 (PMC7526130; doi:10.1186/s12864-020-07080-1)
Supplement: Supplementary file 2 — Additional file 2 : Figure S1. GO classification of assembled contigs. [file 12864_2020_7080_MOESM2_ESM.pdf]

Additional file 2

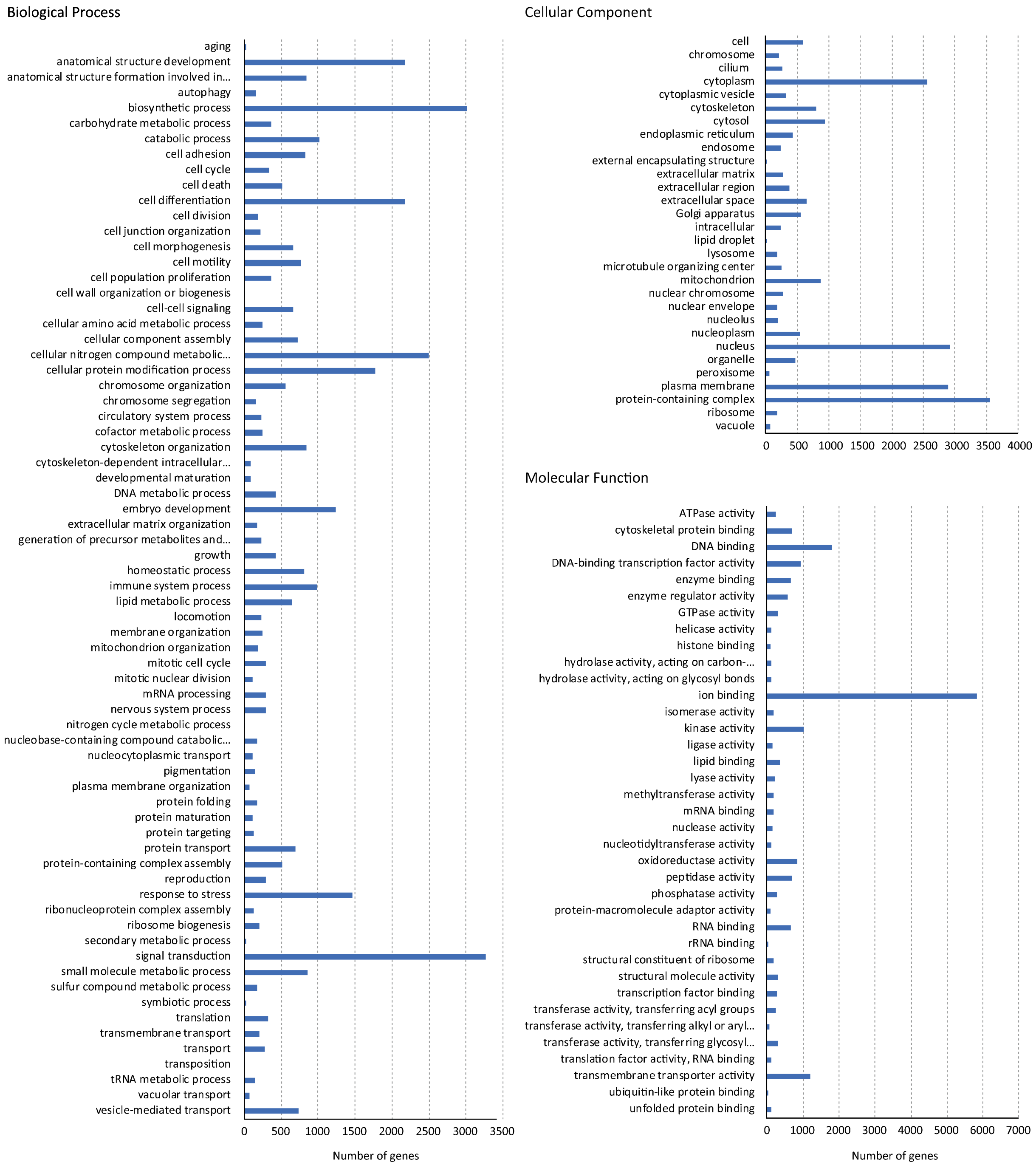

Figure S1

Gene Ontology (GO) classification of assembled contigs.
