# Supplementary material for: Transcriptome characterization of BPG axis and expression profiles of ovarian steroidogenesis-related genes in the Japanese sardine
Source: BMC Genomics. 2020 Sep 29;21:668. doi: 10.1186/s12864-020-07080-1 (PMC7526130; doi:10.1186/s12864-020-07080-1)
Supplement: Supplementary file 4 — Additional file 4 : Sequence alignments of the amino acid sequences of Gth subunits (Figure S2), Gthrs (Figure S3 and S4), and steroidogenic proteins (Figure S5–S9). [file 12864_2020_7080_MOESM4_ESM.docx]

**Additional file 4**

**Fshb**

J. sardine --MHVVAMVA LWSLVGAVVP ECHTQCRLVN TSVSVEME-D GQFCKVETQG CEGLCHNKAP

A. herring -------MAV LWSLVQAVVP DCWTGCQLAN ISVPVETD-D GRSCFIETQA CAGLCHNKDP

Zebrafish MRMRVLVLAL LLPVLMSAES ECRCSCRLTN ISITVESEEC GSCVTIDTTA CAGLCWTMDR

Medakaaaa MQLVVMAAAL VLAEVGQVS- --SFSCHPKN VSIPVES--C GISGCVHTTI CEGRCYHEDP

J. sardine LYWSPVGK-- PRPAEVSCHF EDWIYEMK-- --KCGSGQGQ ---VPVLKAL SCKCSTCDTN

A. herring VYKSSTGVRL PDEQQRSCHF QDSYTETRSF PVHCYTGSSF DLLLTVTKAL SCECSSC-TN

Zebrafish VYPSSMAQ-- --HTQKVCNF KNLMYKSY-- -EFKGCPAGV DSVFVYPVAL SCECNQVNSD

Medakaaaa NYISYEDH-- --PKEKICSG -DWSYEVK-- -FIEGCPVG- ---FKYPVAK SCECTTCNTR

J. sardine QYDCSVISP- GQGCP-PSGH LL---- 128

A. herring KYECDSLSPN MAGCPSPKPP LLQYHL 137

Zebrafish TTDWGAISPQ TTSCSIH--- ------ 130

Medakaaaa TTYCGRLSAD MPSC------ ------ 117

**Lhb**

J. sardine -MARIPQCTI LLFLS----- ----ALVLPS QCF---PCEP FNTTVSVEKL GCPRCLLIQT

A. herring -MARIPECTI LLLLCMC--- ----VLAVPA QCFNLQPCVL VNETVSVEKE GCPRCLVFRT

Zebrafish --MLLAGNGV FFLFSLF--- ----FLLAAA QSLVFPRCEL VNETVSVEKE GCPKCLVFQT

Medakaaaa MISRVSRVMF LLMLSFILGT STFLWSLAPA AAFQLPYCQP VKQKLSLQKE GCSGCHTVET

J. sardine AICHGHCLTK EPVYKSPFSM VSQHVCTYGN LRYETVELPD CDNGVDPVVS YPVALSCECS

A. herring TICSGHCPTK EPVYKSPFSV VNQHVCTYGN FRYETIRLPD CADGVDPLVT YPVALSCECS

Zebrafish TICSGHCVTR DPVYKSPFST VHQTVCMYRD VRYETINLPD CSAGVDPQIT YPVALSCDCS

Medakaaaa TVCSGHCLTK DPLMKIR-SI QYQNVCTYRD FYYKTFELPD CLPGVDPSVT YPVALSCHCG

J. sardine LCSMDTSDCT MGSLEPDYCM GERM---ESQ RLPNYHY 141

A. herring LCSMDTSDCT IESVEPDFCM SQRLPVYESQ KPSLYDY 149

Zebrafish LCTINTSDCT IQSLQPDFCM SQRE------ DFPAY-- 140

Medakaaaa ACIMNASDCT FESLPPDFCV KHDS------ ---FY-- 145

**Cga**

J. sardine ---------- ----MVTQTK SCVASLLLVS ILVHIGDFEM ARD-----CE ECKLKENPIF

A. herring ---------- ----MIIPTK SCVASLLLVS ILVHVGDFYP NSDIATVGCE RCHLGVNDFF

Zebrafish ---------- -----MFWTR YAEASIFLLL MILHVGQLYS RNDVSNYGCE ECKLKMNERF

Medakaaaa MKEKRSHNMT STPPMMGFLK SAEVSVLLMS ILLCTADTYS NLASSNLDCM ECRLEKNSIF

J. sardine SKPGAPVYQC MGCCFSRAYP TPLRSKGTML VPKNITSEAT CCVAREFK-- --SVTFYDVK

A. herring SKPGPQVYQC MGCCFSRAYP TPLRSKNTMM VPKNITSEAT CCVARQYE-- --TVSVYGVN

Zebrafish SKPGAPVYQC VGCCFSRAYP TPLRSKKTML VPKNITSEAT CCVAKESK-- --MVATN-IP

Medakaaaa SREGKPVYQC IGCCFSRAYP TPLRAMQTMT VPKNITSEAT CCVAKHSHEF LFQTIEHTIP

J. sardine LQNHTDCHCS TCYYHKA 114

A. herring VQNHTECHCS TCYHHKS 119

Zebrafish LYNHTDCHCS TCYYHKS 117

Medakaaaa VRNHTECHCS TCYYHKM 137

**Figure S2**

Sequence alignment of the amino acid sequences of gonadotropin subunits (*fshb*, *lhb*, and *cga*). The alignment of protein-coding sequences was performed with Clustal W using BioEdit software. Predictions of N-linked glycosylation were performed using the NetNGlyc 1.0 Server (http://www.cbs.dtu.dk/services/NetNGlyc/). The putative amino acid glycosylation sites are boxed by solid lines. Numbers show the amino acid number. GenBank accession numbers of Fshb, Lhb, and Cga are as follows: Japanese sardine (J. sardine; *Sardinops melanostictus*), LC545605, LC545604, LC545603; Atlantic herring (A. herring; *Clupea harengus*), XP_031413907, XM_031578503, XM_012822755; zebrafish (*Danio rerio*), NM_205624, AAR84283, NM_205687; medaka (*Oryzias latipes*), NM_001309017, NM_001137653, AB541980.

**Fshr**

J. sardine MKSVVPVVML GLSLMGLRGA ANGTLEQPHH CRVNGTLCSF SCLVKEVEKV PTNIPQNTTY

A. herring ---------- ---------- ---------- ---------- ---------M PTDIPKNTTY

Zebrafish ---MVLSMML CFILGCSIAN TEDTLAASQF CAFNGSTRSF ICLGNKVHEI PRRIPTNTTF

Medakaaaa ---MVVMIQM MLVLFRLQMA GASLPETELD DVCFQVELGF S-------TF LRSISSNSTV

J. sardine IVFKLTHICI LPQGAFSNLQ HLKTIVISEN G--------- ---------- -----NLQEI

A. herring MEFKLTRLIA IPKRAFFGLQ HLQRIVVSEN G--------- ---------- -----YLLQI

Zebrafish VEIKLTQISV FRRAALSELH ELKRIVVSEN G--------- ---------- -----ALERI

Medakaaaa VNIKQTQITV IDQSIFTGLW HLEKLTILDN DKLLSICPSA FANLPRLFDM SIQNMALKKI

J. sardine HSLSFANLPS LSEIIITKSK NLVIIYKNAF WNLPKLKHLT ISNTGLKILP VFSGINSFAE

A. herring SDFSFANLSS LTEIIITKSK NLAIIHKNAF WNLPKLKYLS ISNTGLKKLP VFSRINSVVL

Zebrafish EALAFFNLTE LEEITITKSK NL-VMHKDAF WRLPKLRYLT ISNTGLKILP DFSQINSAAL

Medakaaaa GAFAFSNLPA LTEIEITKSR HLTSIHPDAF RSLVGLRRLI ITNTGLRIFP DLSKIHSAVH

J. sardine DFLLDLQENM HLQVIPSNAF QGLSMDTITE LRLTKNGITK VESYAFNGTK MRTLSLMGNQ

A. herring EFLLILQDNM DLKVIPTNAF LGLSMA--SE LRLSNNNITN VESYAFNGTK IQRLSLMGNK

Zebrafish_ EFLFDLQDNM HIERIPSNAF LGLTNATITE LRLTKNGIRE IDSHAFNGTK IGKLFLMGNQ

Medakaaaa RFMFDLQDNI HIQAIPANTF RGLCTQTVEE IRLTRNGIRE VASDAFNGTK MHRLSLKGNK

J. sardine ELILIHRDAF LGAEGPISLD ISYTNVDALP ETMLRTIKSL VATSVYSLRM LPRLDLFADL

A. herring NLSHIDRDAF LGAEGPKSLD ISDTLVDLLP HSMLRTIQSL VAISVKSLRI LPSLKFFPDL

Zebrafish_ QLNHIHSYAF KGAEGPVVLD ISRTAVHTLP ESMLKTLKLL MAVSVYSLRK LPSLELFTEL

Medakaaaa QLTHISGDAF VGSSELVVLD ISETAISSLP DNIISGLKKL LAESAYHLKI LPPPQQFAKL

J. sardine IEANLTYPSH CCAFANFRKN RSERHALCDN PDVSAEE--P ILYRDYCERN TEIICYPKPD

A. herring IEANLTYPSH CCAFADFRKN RSKRLPECDK PNLHREE--P SFYRDNCEEQ TEVICYPKPD

Zebrafish_ TQANLTYPSH CCAFKNFKKH KSVKNQMCNV TGAHEEPDFF NFFNDHCKDV IEVTCYPTPD

Medakaaaa RLAKLTYPSH CCAFKNKPRS RSKWSPLCSH PMAKYIT--- DFYRDHCSNS TSITCSPTPD

ECD

J. sardine AFNPCEDIMG YTYLRVLIWI ICVLAIVGNT VVLLVLLTSR YKLTVPRFLM CHLAFADLCM

A. herring AFNPCEDIMG HTYLRVLIWI ICVLAIVGNS VVLLVLLTSR YKLTVPRFLM CHLAFADLCM

Zebrafish_ AFNPCEDIMG FTFLRVLIWF ISVLAIVGNT VVLLVLLTSR YKLTVPRFLM CHLAFADLCM

Medakaaaa NLNPCEDIMS PVPLRVLIWI ISVLALLGNT VVLVVLLGSR SKLTVPRFLM CHLAFADLCM

J. sardine GIYLMIIASV DIYTRAHYYN FGIDWQMGLG CRAAGFFTVF ASELSVYTLT VITLERWHTI

A. herring GVYLMIIASV DIHTRAHYYN YGIDWQEGVG CKAAGFFTVF ASELSIYTLT VITLERWHTI

Zebrafish_ GIYLLLIAAV DIHTQSRYYN YGIDWQTGAG CHVAGFFTVF SSELSVYTLT AITLERWHTI

Medakaaaa GIYLIIIATI DMLTHGHYYN YAIDWQTGLG CSAAGFCTVF ASELSVFTLT AITVERWHTI

J. sardine TYAMQLERKL RMRHAAALMA GGWAFAWLAA LFPAVGIASS YMKVSICLPM DVKSAASQTY

A. herring TYAMQLERKL QLRHAAALMA GGWAFAWLVA LLPALGLSSS YKEVSICLPM DVEKATSQIY

Zebrafish_ TYAMQLERQM RLRHACLVMA TGWLFSLLTA LTPMFGVSS- YSKTSICLPM DVETLLSQGY

Medakaaaa THALRLDRKL RMRHACVIMA AGWIFSSLAA LLPTVGVSS- YSKVSVCLPM DVESLVSQVY

J. sardine VVLLLLLNVA AFFVVCACYL RIYTTVHNPR ALAPLTSASD MRLAKRMAVL IFTDFLCMAP

A. herring VVLVLLLNVS AFLGMCACYL GIYTTVHNPH ALAPLTSASD TRLAKRMAVL IFTDFLCMAP

Zebrafish_ VVLLLLLNAA AFLVVCVCYT LIYLTVRNP- AFVPAN--AD MRIAKRMAVL IFTDFLCMAP

Medakaaaa LVSLLLLNIL AFFCVCGCYL SIYLTYRKP- SSAPAH--AD TRVAQRMAIL IFTDFLGMAP

J. sardine ISFFAISAAF KWPLINVSQA KVLLVLFYPI NSCANPFLYA FFTKTFKRDF FVLASRFGCF

A. herring ISFFAISAAL KWPLITVSHS KVLLVLFYPI NSCANPFLYA FFTKTFKRDF FVLASRFGCF

Zebrafish_ ISFFAISAAF KLPLITVSHA KVLLVLFYPI NSCSNPFLYA FFTKTFKRDF FILTSRFGCF

Medakaaaa VSFFAISAAL KLPLITVSDS KLLLVLFYPI NSYSNPFLYA FFTRTFRRDF FLLAARFGLF

J. sardine KMQAQIYRTE SSSVQNGVWV PSPKTSETTI YSLVHMTHKY ----- 674

A. herring KMQAQIYRTE SS-VQNGVWV NSPKSSEGTI YSLVHMTYKH ----- 622

Zebrafish_ KRRAHIYRTE ISSGQNGAVV PSPKTSDGTL YSLVHIAQVH ----- 668

Medakaaaa KTRAQIYRTE TSSCQQPTWT -SPKSSR-VM YSLANTLSLD GKQEC 686

**Figure S3**

Sequence alignment of the amino acid sequences of follicle-stimulating hormone receptor (*fshr*). ECD, extracellular domain. Numbers show the amino acid number. GenBank accession numbers are as follows: J. sardine, LC545601; zebrafish, AY278107; medaka, NM_001201514. For A. herring, since the latest *fshr* sequence (XP_031438581) does not contain full-length CDS, the other full-length CDS sequence (XP_012679228) was retrieved from RefSeq assembly accession, GCF_000966335.1.

**Lhr**

J. sardine ---------M MWIFHLLPVF CVWLHCESVF ASSYECPPMC ECYINSIRCN NRTEHAATLT

A. herring ---------- MWIFHLLPVL YVWLKCESVF ASPFTCPQIC VCSINSIRCN NKTEHGATLT

Zebrafish_ ---------- MWRSALLLVF --LLLTSFCC GVCFECPEIC RCSQKSITCN SATESQKSLS

Medaka MSCGPPARMA PRVVWLLVAL SGVLNARSCQ A--YPCHPIC RCTPDTFQCN RGTQLAAGTA

J. sardine QKKPGKDINL MYLSLKVISS HSFDGLREVM SILITQSITL ERIEPLAFNS LYNLTDITVK

A. herring QKKACINLNL QFISLKDIGS RSFDGLRDVK RIRITHSVTL ESIQPKAFNN LHNLSEILIQ

Zebrafish_ R------LVL NYISVKTISS RSFDGLKGVR RIEIAQSSSV ETIESEAFNN LPNVSEISIQ

Medaka EHR----LRL THLPLKQVPT HAFKELMNIT IIEISQSDRI TAIQRHAFLS LHSLQQILVL

J. sardine NTRNLVFIGQ RAFNNLPKLK HLVISNTGIT VFPDFSAVSS LNPSFELLEM CDNLLLTSIP

A. herring NTRSLVHIGR RAFNHLPNLQ YLCISNTGIT VFPDLTALSS LNPSF-VLDI FDNLLLTSIP

Zebrafish_ NTRNLVHIQQ RAFNQLPKLR YLSISNTGIS VFPDLTSIFS LEAHF-ILDI CDNLNLRSVP

Medaka NINSLRGIER GAFTDLPRLE FLTISNTGMM HFPDFTSVSS LTPSI-LLEM LDNMRIDVIP

J. sardine PNAFLGIAIS --TMNLYNNG FKEIQSHAFN GTVIEQLSLK YNRDLRVIHR EAFTGAVGPV

A. herring ANAFLGMATS --TMNLYNNG FKEIQSHAFN GTVIDKLILK NNKNLRVIHR EAFTGAVGPT

Zebrafish_ SNAFTGMTSE YATMNLFNNG FQEIESHAFN GTKIDKLVLK NSRDLRVIHE DAFKGALGPT

Medaka ANSFRGMTKG HANMNLVRNG FKEIQSHAFN GTKLNNLILR DNRFLSYIAE DAFEEATGPS

J. sardine LLNVSSSVLE TLPPHGLESV EVLSAEKAFA LKRLPNFNSL PSLRDARLTY PSHCCALNSW

A. herring LLDVSSTGLD TLPPRGLESV GVLMAQAAFA LKRLPNFNSL RSLWSASLTY PSHCCALHSW

Zebrafish_ VLGVSSTALE TLPSHGMESV LMLTARSAFA LKKLPPLKSL KSLREAQLTF PSHCCALINW

Medaka YLDVSSTALS ALPAKGLTRV QTLKATATFA LKSLPPLQSL AELLEAELTY PSHCCAFDKW

J. sardine HAHRES---- LLSNGSIS-- ---------- -WDIS----- ------EAEA L----EHYPD

A. herring NAHRESSLIA AVNNGSVSCE EDTPSAGVHG SWSLEPPV-- ISDMTFEEET FGSVDSLYPE

Zebrafish_ DNSRDGSVNS ALRNRSSYCG DNSSPADLSA ISSDDTLESD VIGSSSVEDT FGSIDFHYPD

Medaka RRKQREN--- ALKNSTKLCN --------LG ETEIEATD-- -----DGMNL VNDIKFEYPD

ECD

J. sardine LDPER---LP LKCTPEPDAF NPCEDLVGFG FLQVAIWLIN ILAIIGNLTV LLVFFTSGTK

A. herring LDLFRR-KTA LTCNPEADAF NPCEDLAGFG FLRVAIWFIN ILAIVGNLTV LLVFFTSGTK

Zebrafish_ LDLCQQ-RQA LQCSPEADAF NPCEDIAGFS FLRVAIWFIN ILAIAGNLVV LLVLFTSRCK

Medaka LEFDCGSNPF VICTPTPDDF NPCEDLLRYA FLRCLTWIIT IFAVAGNLAV LVILLVSHHK

J. sardine LTVPRFLMCH LAFADLCIGI YLLMIAAVDL RTRGLYSQHA IEWQTGAGCS VAGFLAVFGG

A. herring LTVPRFLMCH LAFADLCIGV YLLMIAAVDL RTRGHYSQHA IEWQTGTGCS VAGFLAVFGG

Zebrafish_ LTVPRFLMCH LAFADLCIGI YLLMIATVDL RTRGHYSHHA IEWQTGAGCG IAGFLSVFGG

Medaka LTISRFLMCN LAFADLCMGL YLMLIAFMDF HSRHEYYNHA TDWQTGPGCG TAGFLTVFAS

J. sardine ELSVYTLCNI TLERWHTITH AMRLERRLGL SHASAIMAAG WLLCLGVALL PLVGVSSYSK

A. herring ELSVYTLCTI TLERWHTINH ALHLERRMGL SHAAAIMAAG WLLCLGVALL PLVGVSSYSK

Zebrafish_ ELSIYTLSTI TVERWHTITH ALRLERRLGL SQASLIMTIG WLLCLAMALL PLIGVSSYSK

Medaka ELSVYTLTVI SIERWHTITN AMHVNKRLRM HHVTAMMAAG WVFSLLVALL PLVGVSSYSK

J. sardine VSMCLPMDIE TPLAQAFVIL LLLLNVAAFL VVCVSYVRIY VAVHNPEFPG RNADTKIAKR

A. herring VSMCLTMDIE TALAQAFIIL LLLFNVGAFL AVCVCYARIY VAVHNPEFPG RSADTKIAKR

Zebrafish_ VSMCLPMDIE TPLSQAYVIL LLLFNVGAFL VICGCYVCIY SAVRNPEFPG RAADAKIAKR

Medaka VSICLPMDID TLSSQVYVVT FLILNVAAFL VVCFCYIGIY VSVRNPEHST RNGDTKIAKR

J. sardine MAVLIFTDFL CMAPISFFAI SAAFKVPLIT VTNSKILLVL FYPINSCANP FLYAIFTKAF

A. herring MAVLIFTDFL CMAPISFFAI SAAFKVPLIT VTNSKILLVL FYPINSCANP FLYAICTKAF

Zebrafish_ MAVLIFTDFL CMAPISFFAI SAAFKVPLIT VTNSKILLVL FYPINSCANP FLYAIFTRAF

Medaka MAVLIFTDFL CMAPISFFAI SAALRMPLIT VSHSKILLIL FYPINSLCNP FLYTISTRAF

J. sardine RKDAYKFLSS MGCCKSKAGL YRMKRYCSGK IAQNRSDGG- ---HKGP-GV RHLSAFQHQK

A. herring RKDACKLLSS MGCCEIKASL YRMKTYCSGS MAKSHSDGG- ---AKRLRGG QDPSSFQHQK

Zebrafish_ RKDACILLSS MGCCQSKANL YRMKTYCSEN INRSKSSSGS NANSKGPRAV MWMSSFPQLT

Medaka RKDVCRLASR CSCCRANANS CRSKASQQAC ARRVT----- ---SQKPHSL NFYAYHIKMK

J. sardine PQIETQSTEG 679

A. herring QLKSEAKGKD 711

Zebrafish_ PRPHIQRV-- 708

Medaka GCFLSEGAT- 696

**Figure S4**

Sequence alignment of the amino acid sequences of luteinizing hormone receptor (*lhr*). Numbers show the amino acid number. GenBank accession numbers are as follows: J. sardine, LC545602; A. herring, XM_031579220; zebrafish, AY714133; medaka, NM_001201515.

**Cyp11a1**

J. sardine MARWSVRLSS QARALVDSAR ---VRVRHSS SVPAAREAAY PECSSSVRPF KELPGTWKNG

A. herring MARWSVCLR- -ARSLVESAR ---VRACHSG SMPAAREAAY PQLTSAIRPF KEIPGSWKNE

Zebrafish_ MARWNVTLAR LDQSLSSLKN LLQVKVTRSG RAPQ------ ---NSTVQPF NKIPGRWRNS

Medaka MARWHMCRST VGLPLSWAEE PIASGARSSS SMPVDSGSIP GEQQRCAGLS MRFPDRWKNG

J. sardine VVTVYNFWKL DGFKNIHNIM IHNFNTFGPI YREKIGSYDS VNIINPEDAA ILFKAEGHYP

A. herring VATLYNFWKQ DGFKNIHNIM VNNFNTFGPI YREKIGYYDS VNIINPEDAA ILFKAEGHYP

Zebrafish_ LLSVLAFTKM GGLRNVHRIM VHNFKTFGPI YREKVGIYDS VYIIKPEDGA ILFKAEGHHP

Medaka LVNLYNFWKL DGFKNLHRIM VQNFNTFGPI YREKIGYYES VNIIKPEDAA TLFKAEGHYP

J. sardine KRFRVEPWTS YRDYRHREYG VLLKDGEDWR SNRVVLNKEV IAPKVQGNFV PLLDEVGQDF

A. herring ERFRVEPWIA YRDFRHRERG VLLKDGEDWR SNRVVLNKEV IAPRVQGNFV PLLDEVGQDF

Zebrafish_ NRINVDAWTA YRDYRNQKYG VLLKEGKAWK TDRMILNKEL LLPKLQGTFV PLLDEVGQDF

Medaka KRLKVEPWTS YRDYRNRKYG VLLKNGADWR SNRMILNKEV IFLKMLENFV PLLDDVGQDF

J. sardine VARIYKKIER SGQSKWTIDL QQELFKYALE SVGSVLYGER FGLLQDHIDP EAKRFIDSIT

A. herring VARIYKKIER SGQNKWTVDL HQELFKYALE SVGSVLYGER FGLLQDHIDP DAQRFIDSIT

Zebrafish_ VARVNKQIER SGQKQWTTDL THDLFRFSLE SVSAVLYGER LGLLLDNIDP EFQHFIDCVS

Medaka VARVHKKIKR NGQNKWTTDL SQELFKYALE SVGSVLYGER LGLLLDYIDP EAQHFIDCIT

J. sardine LMFKTTTPML YIPPGLLRRL GSRVWREHVE AWDTIFNQAD RCIQNIYREL RTSKKEDTVD

A. herring VMFKTTTPML YIPPALLRRL GSRVWRDHME AWDVIFNQAD RCIQNIYRQL RAGK-EDTLD

Zebrafish_ VMFKTTSPML YLPPGLLRSI GSNIWKNHVE AWDGIFNQAD RCIQNIFKQW KENP----EG

Medaka LMFKTTSPML YIPPSVLRRI GSKVWRDHVD AWDGIFNQAD RCIQNIYREL RLET----GT

J. sardine QGKYPGVLAN LLMMDKLSIE DIKASITELM AGGVDTTSTT LLWTLYELSR NTDLQEELRA

A. herring QNKYPGVLAN LLMLDKLSIE DIKASVTELM AGGVDTTSTT LLWTLYELSR NPDLQEELRA

Zebrafish_ NGKYPGVLAI LLMQDKLSIE DIKASVTELM AGGVDSVTFT LLWTLYELAR QPDLQDELRA

Medaka SEKYPGVLAS LLMLDKLSIE DIKASITELM AGGVDTTSIT LLWTLYELAR HPSLQEELRL

J. sardine EVLSARHSSQ GDLLLMLKSV PLLKAAIKET LRLHPVAVNL QRYITEDIVM QNYLIPAGTL

A. herring EVLSAQQTSQ GDILLMLKSL PLLKAAIKET LRLHPVAVSL QRYITEDTVM QNYQIPAGTL

Zebrafish_ EISAARIAFK GDMVQMVKMI PLLKAALKET LRLHPVAMSL PRYITEDTVI QNYHIPAGTL

Medaka EVAAARARSQ GDMMEMLKRV PLIKGAIKET LRLHPVAVSL QRYITEDIVI QNYHIPAGTL

J. sardine VQLGLYAMGR SHRIFPHPEV YRPSRWLRTE SQYFRSLGFG FGPRQCLGRR IAETEMQLFL

A. herring VQLGLYAMGR NHRIFPHPET YQPSRWLKRD TAYFKSLGFG FGPRQCLGRR IAETEMQLFL

Zebrafish_ VQLGVYAMGR DHQFFPKPEQ YCPSRWISSN RQYFKSLGFG FGPRQCLGRR IAETEMQIFL

Medaka VQLGLYAMGR DPKVFFRPEQ YQPSRWLRTE THYFRSLGFG FGPPQCLGRR IAETEMQIFL

J. sardine IHMLENFRIE KQSKVKVQST FELILVPERP IVLNIRPLNS TQ 519

A. herring IHMLQNFRIE RQNKVKVKSK FELILVPDKP IVLTIRPLSS GQ 516

Zebrafish_ IHMLENFRIE KQKQIEVRSK FELLLMPEKP IILTIKPLNA SR 509

Medaka IHMLENFRVE KQRHIEVQST FELILLTDKP IILTLKPLQA NP 518

**Figure S5**

Sequence alignment of the amino acid sequences of cytochrome P450 side-chain cleavage (*cyp11a1*). Numbers show the amino acid number. GenBank accession numbers are as follows: J. sardine, LC545596; A. herring, XM_031563038; zebrafish, AF527755; medaka, NM_001163086.

**Cyp17a1**

J. sardine ------MAWL MSTFLFPAFV VILYFLKRRF GAS---QNKS PPALPSLPII GSLLSLKTDR

A. herring ------MAWL ICTCLFSAFM MILYFLKRRV VAFR-LGKKS PPALPSLPII GSLLSLKSDR

Zebrafish_ MAEALILPWL LCLSLFSAVT LAALYLKQKM NGFVPAGNRS PPSLPSLPII GSLMSLVSDS

Medaka ------MAWF LCLSVLVVLV LALAALLWRV RTRD--RPQE APSLPYLPVL GSLLSLRSPH

J. sardine PPHIFFQELQ KKYGDTYSLM MGSHNVVIVN SHQHAREVLM KKGKTFAGRP RTVTTDILTR

A. herring PPHIFFQELQ KKYGDTYSLK LGSHNIIIVN SHQHAKEVLI KKGKTFAGRP RTVTTDILTR

Zebrafish_ PPHIFFQDLQ KKYGDLYSLM MGSHKLLIVN NHHHAKEILI KKGKIFAGRP RTVTTDLLTR

Medaka PPHVLFKELQ QKYGQTYSLK MGSHQVIIVN HHAHAREVLL KRGRTFAGRP RTVTTDVLTR

J. sardine DGKDIAFADY SPTWKFHRKI VHGALCMFGE GTASIEKIIC REAESMCKTL GEMQSL--AV

A. herring GGKDIAFADY SPTWKFHRKI VHGSLGMFGE GTASIEKIIC READAMCKTL GEMQSL--AV

Zebrafish_ DGKDIAFADY SSTWKFHRKM VHGALCMFGE GSVSIEKIIC REASSMCEVL TESQNS--AV

Medaka DGKDIAFGDY SATWRFHRKI VHGALCMFGE GSASLQRIIC TEAQSLCSTL SEAAATGLAL

J. sardine DLAPELTRAV TNVVCSLCFS SSYKPGDQEF EAMLEYSQGI VDTVAKDSLV DIFPWLQIFP

A. herring DLAPELTRAV TNVVCSLCFS SSYKRGDQEF EAMLDYSQGI VDTVAKDSLV DIFPWLQIFP

Zebrafish_ DLGPELTRAV TNVVCALCFN SSYKRGDAEF ESMLQYSQGI VDTVAKDSLV DIFPWLQIFP

Medaka DLSPELTRAV TNVICSLCFN SSYSRGDPEF EAMLRYSQGI VDTVAKDSLV DIFPWLQIFP

J. sardine NKDLKILRDC VSIRDKLLQK KYEEHKTNYS DNIQGDLLDA LLRAKRSSEN NNT----STQ

A. herring NKDLNILRDC VSIRDRLLQK KYEEHKTNYS DNVQGDLLDA LLRAKRSSEN NNT----TTQ

Zebrafish_ NKDLRILRQC ISIRDKLLQK KYEEHKVTYS DNVQRDLLDA LLRAKRSSEN NNS----STR

Medaka NKDLRLLKQC VAVRDQLLQK KFEEHKSDYS DHVQRDLLDA LLRAKRSAEN NNTAAEFSAE

J. sardine GVGLTDDHVL MTVGDIFGAG VETTTTVLKW TIAYLIHHPQ VQAKIQAELD RKIGKDRHPL

A. herring DVGLTDDHLL MTVGDIFGAG VETTTTVLKW SIAYLIHHPQ VQAKIQAELD RKIGKERHPQ

Zebrafish_ DVGLTEDHVL MTVGDIFGAG VETTTTVLKW SIAYLVHNPQ VQRKIQEELD SKIGKERHPQ

Medaka AVGLSDDHLL MTVGDIFGAG VETTTTVLKW AITYLIHYPE VQKQIQEELD RKVGVDRPPQ

J. sardine LSDRGNLPYL EATIREVLRI RPVSPLLIPH VATTDASIGE YTVKRGTRVI VNLWSLHHDQ

A. herring LSDRGNLPYL EATIREVLRI RPVSPLLIPH VAMADASIGD FTVKRGARVI VNLWSLHHDQ

Zebrafish_ LSDRGNLPYL EATIREVLRI RPVSPLLIPH VALQDSSVGE YTVQKGTRVV INLWSLHHDE

Medaka LSDRGSLPFL EATIREVLRI RPVAPLLIPH VALSDTSLGD FTVRKGTRVV INLWSLHHDE

J. sardine KEWKNPDLFD PGRFLDEEDG GPCCPSASYL PFGAGVRVCL GEALAKMELF LFLSWILQRF

A. herring NEWKNPELFD PGRFLDKEGA GLCSPSASYL PFGAGVRVCL GEALAKMELF LFLSWILQRF

Zebrafish_ KEWKNPELFD PGRFLNEEGD GLCCPSGSYL PFGAGVRVCL GEALAKMELF LFLAWILQRF

Medaka KEWTNPDLFN PGRFLSADGS SLTLPSSSYL PFGAGLRVCL GEALAKMELF LFLSWILQRF

J. sardine SLEVPAGHPL PSLEGKFGVV LQPQKYKVLA RLREGWEKGQ ALSTEE 511

A. herring TLEVPVGHPL PSLEGKFGVV LQPQKYKVLA RLREGWEKGQ AFSPEE 513

Zebrafish_ TLEMPTGQPL PDLQGKFGVV LQPKKFKVVA KVRADWEKSP LMQHC- 519

Medaka TLSVPPSQSL PSLEGKFGVV LQPVKYAVKA TPRPGCHSGL FPAN-- 516

**Figure S6**

Sequence alignment of the amino acid sequences of 17α-hydroxylase/C17,20-lyase (*cyp17a1*). Numbers show the amino acid number. GenBank accession numbers are as follows: J. sardine, LC545597; A. herring, XM_012840404; zebrafish, NM_212806; medaka, NM_001105094.

**Cyp19a1a**

J. sardine MAADLLQPCM HAMDQLHMEQ TMTELWFSRP LNATQSLPES LSGTTA-ALL IMLGLLVAL-

A. herring MAAKLLQPCL HTIDQLHMDQ TMMELWIAGP LNMTNSLPEK VSGTTS-TLL LIICLLVAF-

Zebrafish_ MAGDLLQPCG --MKPVRLGE AVVDLLIQRA HNGTERAQDN ACGATATILL LLLCLLLAIR

Medaka --MDLIPACD RTMSSS---- CLVAELVSIA PNTTVGLPSG IPMATR-SLI LLVCLLLMVW

J. sardine LNLNDKSSVP GPSFYLGLGP LLSYSRFIWT GIGTASNYYN KQYGDIVRVW INGEETLILS

A. herring FNR-KTTSVP GPSFNLGLGP LLSYSRFIWT GIGTASNYYN KKYGDIVRVW INGEETLILS

Zebrafish_ HHRPHKSHIP GPSFFFGLGP VVSYCRFIWS GIGTASNYYN SKYGDIVRVW INGEETLILS

Medaka SHS-EKKTIP GPSFCLGLGP LMSYLRFIWT GIGTASNYYN NKYGDIVRVW INGEETLILS

J. sardine RSSAVYHVLK QSKYTARFGS KQGLQCIGMH DRGIIFNSNV TLWKKVRAFY AKALTGPGLQ

A. herring RSSAVYHVLK QSKYTSRFGS KQGLQCIGMD ERGIIFNSNV TIWKKVRTFF AKALTGPGLQ

Zebrafish_ RSSAVYHVLR KSLYTSRFGS KLGLQCIGMH EQGIIFNSNV ALWKKVRAFY AKALTGPGLQ

Medaka RASAVHHVLK NRKYTSRFGS KQGLSCIGMN EKGIIFNNNV ALWKKIRTYF TKALTGPNLQ

J. sardine KTLEICASST LKQLEQLQHL SSPEGRVDVL TLLRCVVVDI SNRLFLGVPF NEKELLSKIH

A. herring RTLEICMSST NKHLEQLEQL SSPEGHVDVL NLLRCVVLDI SNRLFLGVPF DEKELLFKIQ

Zebrafish_ RTMEICTTST NSHLDDLSQL TDAQGQLDIL NLLRCIVVDV SNRLFLGVPL NEHDLLQKIH

Medaka QTVEVCVTST QTHLDNLSSL S----YVDVL GFLRCTVVDI SNRLFLGVPV DEKELLQKIH

J. sardine NYFETWQTVL IKPDIYFKLE WLHNKHKRAA QELQDTIESL IEKKRIMLRS AEKLDNIDFT

A. herring KYFDTWQTVL IKPDIYFKFE WLHNKHKRAA QELQDTIESL IEEKRAMLGS TEKLDTIDFT

Zebrafish_ KYFDTWQTVL IKPDVYFRLD WLHKKHKRDA QELQDAITAL IEQKKVQLAH AEKLDHLDFT

Medaka KYFDTWQTVL IKPDIYFKFS WIHQRHKTAA QELQDAIESL VERKRKEMEQ AEKLDNINFT

J. sardine TDLIFAQNHG ELSADNVRQC VLEMVIAAPD TLSISLFFML LLLKQHPEVE KKILDELDTM

A. herring TDLIFAQSRG ELTAENVRQC VLEMVIAAPD TLSISIFFML LLLKQHPEVE KKILEELDTM

Zebrafish_ AELIFAQSHG ELSAENVRQC VLEMVIAAPD TLSISLFFML LLLKQNPDVE LKILQEMDSV

Medaka AELIFAQGHG ELSAENVRQC VLEMVIAAPD TLSISLFFML LLLKQNPHVE LQLLQEIDTI

J. sardine IGDKPLRHAD LPKLTVLESF INESLRFHPV VDFTMRRALA DDVIDGYKVT KGTNIILNVG

A. herring IGDKYLRHAD LQKLPVLESF INESLRFHPV VDFTMRRALA DDVIDGYKVS KGTNIILNTG

Zebrafish_ LAGQSLQHSH LSKLQILESF INESLRFHPV VDFTMRRALD DDVIEGYNVK KGTNIILNVG

Medaka VGDSQLQNQD LQKLQVLESF INECLRFHPV VDFTMRRALF DDIIDGHRVQ KGTNIILNTG

J. sardine RMHKTEFFLK PNEFSLDNFD KNVPNRFFQP FGSGPRSCVG KHIAMVMMKS ILVTLLSRYS

A. herring RMHKTEFFKK PNEFSLDNFD ENVPNRFFQP FGSGPRSCVG KHIAMVMMKS ILVTLLSRYS

Zebrafish_ RMHRSEFFSK PNQFSLDNFH KNVPSRFFQP FGSGPRSCVG KHIAMVMMKS ILVALLSRFS

Medaka RMHRTEFFHK ANEFSLENFQ KNTPRRYFQP FGSGPRACVG RHIAMVMMKS ILVTLLSQYS

J. sardine VCPRRDCTVE NIPQTNNLSQ QPVEE--DEA SMMVGFIPRK R-LRSNPGKA 525

A. herring VCPRHDCTVE NIPQTNNLSQ QPVEV--DDA PMMVRFIPRK R-LRSNPGKA 524

Zebrafish_ VCPMKACTVE NIPQTNNLSQ QPVE---EPS SLSVQLILRN T-L------- 517

Medaka VCPHEGLTLD CLPQTNNLSQ QPVEHHQEAD HLSMTFLPRQ RGIWESPSPF 518

**Figure S7**

Sequence alignment of the amino acid sequences of aromatase (*cyp19a1a*). Numbers show the amino acid number. GenBank accession numbers are as follows: J. sardine, LC545598; A. herring, XM_012814772; zebrafish, NM_131154; medaka, NM_001278879.

**Hsd3b**

J. sardine MSLSGDVCVV TGACGFLGQH LVKLLLEEEK LAEIRLLDRN VQPELIQELE DKRGETTVSV

A. herring MSLSGDVCVV TGACGFLGKN LVQLLLEEEK LAEIRLLDKT VQPELVRDLE DKRGETTLSF

Zebrafish_ MALSGEVCVV TGACGFLGER LVRLLLKEEK LAEIRLLDRN IRSELIQSLD DCRGETKVSV

Medaka MSLRGDVCVV TGACGFLGKR LVRLLLEEEE TAEIRLLDKH VPQQVLQSLE DCRGGTKLSA

J. sardine FEGDIRDTDL LRRACRGASI VFHTVSLIDV IGTLKYSELH EVNVKGTQML LEACVQENVC

A. herring FEGDIRNADL LRRACQGASI IFHTASLIDV TGRVDESELH GVNVKGTQML LETCIQENVC

Zebrafish_ FEGDIRNPEL LRRACKGAAL VFHTASLIDV IGAVEYSELY GVNVKATKLL LETCIQENVP

Medaka FEGDIRDSDF VRKACRGATN VFHTASMIDV LESVEYSEIY GVNVKGTQLL LEACLHENVM

J. sardine SLIYTSSIEV AGPNPRGDPV VNGDEDMPYS ACLKFPYSRT KKEAEDLCLR AQGEVLRNGG

A. herring SIIYTSSIEV AGPNPHGDPI FNGDEETPYS ACLKFPYSRT KKEAEELCCR AQGEALRNGG

Zebrafish_ SFIYTSSIEV AGPNPSGEPI INGNEDTPYS SRLKFSYSKT KKEAEEICLQ ANGDLLCNGG

Medaka SFIYTSTIEV VGPNPRGDPM VNGTEDTVYD SRLTLSYSKT KKEAEDRTLQ ANGQLLQNGG

J. sardine RLATCALRPM YIFGAGCRFT LGHMRDGVRN GDVLLRASPR QALVNPVYVG NVAAAHLQAA

A. herring RLATCTLRPM YIFGAGCRFT LGHMRDGIHN GDVLRRMSPR KALVNPVFVG NVAFAHLQAA

Zebrafish_ QLATCALRPM YIFGPGCRFT LGHMRDGIRN GNVLLRTSRR EAKVNPVYVG NAALAHLQAG

Medaka RLATCSLRPA YIFGEGCRFL LGHMTDGIRK GNVLNRLSAR EALVNPVYVG NVAFAHLQAA

J. sardine RALRDPRKRA VVGGSMYYVA DDTPPVSYSD FNHAVLAPLG FGIQERPALP FPVLYVVCFL

A. herring RALRDPRRRA LVGGNFYYVA DDTPPVSYSD FNHVVLAPLG FDIQERPTLP FPVLYLLCFL

Zebrafish_ RGLRDPQKRA MMGGNFYYIS DNTPHVSYSD FNYAVLSSLG FGIQERPILP FPLLYILSFF

Medaka RSLKEPQKRD TVGGKFYFIT DDTPHLSYAD FNYCMMSPLG FSVQDKLQMP LRIFYIVIFF

J. sardine MEMLQTVLRP FLRFAPPLNR QLLTMLYTPF TFSYQKAKRD LGYTPRYSWE EARKTTTDWL

A. herring MEMLQMMLRP FVRFTPPLNR QLLTMLNTPF SFSYQKAKRD LGYTPRYGWE EARKITTDWL

Zebrafish_ MELLHVVLRP FLTFTPPLNR QLLTMLNTPF SFSYQKAHRD FGYTPRYEWE EARKCTTDWF

Medaka LEALCMLLRP FIRIVPPMNR QLLTLLNTTF TFSYQKAKRD LGYFPKYSWE EARRRTFDWL

J. sardine ALMLAKERAQ VKVK 374

A. herring ALMLAKERAH MKAK 374

Zebrafish_ ASVLPAEIRS INLK 374

Medaka ASQLPQQRET LQSN 374

**Figure S8**

Sequence alignment of the amino acid sequences of 3β-hydroxysteroid dehydrogenase (*hsd3b*). Numbers show the amino acid number. GenBank accession numbers are as follows: J. sardine, LC545599; A. herring, XM_012842327; zebrafish, XM_689112; medaka, NM_001137565.

**Star**

J. sardine MFPATFKLAA GISYRHMRNM TGLQKNAVVA IQHKFRSLSG PGPSTWISHV RRRSSLRSTR

A. herring MFPATFKLCA GISYRHMRNM TGLRKNAMVA IHHELSRLSC PGPSSWISNV RRRSSLRSNR

Zebrafish_ MLPATFKLCA GISYRHMRNM TGLRKNAMIA IHHELNKLSG PGASTWINHI RRRSSLLSSP

Medaka MLPATFKLCA GISYRHMRNM TGLRKNAIVA IHHELNRLAG PGPSNWISQV RRRSSLLSSR

J. sardine IEEKPACSET DLAYVKQGQE ALQKSISILS EQDGWQTEII SASGDRVLSK VLPDIGKVFK

A. herring IEEEP-CSEV DLAYVKQGQE ALQKSICILS EQNGWQTEIV TASGDKVLSK VLPDIGKVFK

Zebrafish_ IAEET-YSEA DQCYVQQGQE ALQKSISILE DQDGWQTEIE SINGEKVMSK VLPGIGKVFK

Medaka IEEEG-YSEE EMSYVKQGED ALQKAISILS EQDGWTVETV APNGDKVLSK VLPDIGKVFK

J. sardine LEVVLEQHPD SLYNELVGNM EQMGDWNPNV KQVKILQKIG EDTMVTHEVS AETPGNVVGP

A. herring LEVVLDQHPD DLYNELVGNM EQMGDWNPNV KQVKILQKIG QDTMVTHEVS AETPGNVVGT

Zebrafish_ LEVTLEQQTG DLYDELVDNM EQMGEWNPNV KQVKILQKIG QETMITHEIS AETPGNVVGP

Medaka LEVVMEQHPD SLYEELVGNM EQMGEWNPNV KEVKILQKIG PDTMITHEVS AETPGNVVGP

J. sardine RDFVSVRCAK RRGSTCFLAG MSTQHPGMPE QKGVVRAENG PTCIVMRPSA EDPDKTKFTW

A. herring RDFVSVRCAK RRGSTCFLAG MSTQHPGMPE QKGVVRAENG PTCIVMRPNA DDPEKTKFTW

Zebrafish_ RDFVNVRHAK RRGSTCFLAG MSTQHPGMPE QKGFVRAENG PTCIVMRPSA DDPNKTKFTW

Medaka RDFVNVRCTQ RRGSTCFLAG MSTQHPKMPE QKGIIRAENG PTCIVMKPCA EHPNKTKFTW

J. sardine LLSIDLKGWI PKTIINRVLS QTQLDFANHL RHRMTVSGGL EVAALAC 287

A. herring LLSIDLKGWI PKSIINRVLS QTQLDFANHL RTRMGPSGGL E-AALAC 285

Zebrafish_ LLSLDLKGWI PKTVINRVLS QTQVDFVNHL RDRMASGGGI D-AAIAC 285

Medaka LLSLDLKGWI PKTIVNKVLS QTQVDFANYL RHRMADNVSS E-LAPAC 285

**Figure S9**

Sequence alignment of the amino acid sequences of steroidogenic acute regulatory protein (*star*). Numbers show the amino acid number. GenBank accession numbers are as follows: J. sardine, LC545600; A. herring, XM_012815859; zebrafish, NM_131663; medaka, NM_001104910.
