# Supplementary material for: Transcriptome characterization of BPG axis and expression profiles of ovarian steroidogenesis-related genes in the Japanese sardine
Source: BMC Genomics. 2020 Sep 29;21:668. doi: 10.1186/s12864-020-07080-1 (PMC7526130; doi:10.1186/s12864-020-07080-1)
Supplement: Supplementary file 5 — Additional file 5 : Molecular characterization of Japanese sardine Fshb. Figure S10. Sequence alignment of the amino acid sequences of Japanese sardine Fshb isoforms. [file 12864_2020_7080_MOESM5_ESM.doc]

**Additional file 5**

Molecular characterization of Japanese sardine follicle-stimulating hormone subunit beta (Fshb)

**Results and Discussion**

In the present study, multiple clones exhibiting different amino acid sequences for Fshb were obtained from ten pituitaries. Individual fish had one or two types of Fshb. Figure S10 shows the sequence alignment of Fshbs obtained from the Japanese sardine. The predicted locations of cysteine-knot loops (βloop-1 to -3) and seatbelt loop are also presented. All isoforms contained 12 cysteine residues and the region of cysteine residues is conserved. As compared to the main sequence (isoform 1), substitutions of amino acids were found in the region of loop-3, where amino acid residues at six positions (Met84, Lys86, Gly90, Lys91, Gly92, and Gln 93) were substituted or lacking (isoforms 2, 3, and 5). Of these isoforms, some isoforms were accompanied by additional substitutions in the C-terminal domain, where amino acid residues at seven positions (Val116, Ile117, Gln121, Gly122, Pro124, Ser126, and His127) were substituted (isoform 3). In the main sequence (isoform 1), one potential N-glycosylation site was located at βloop-1. A shift of a single N-linked glycosylation site (NTS) at Asn30 to Asn32 (NVS) was found in the amino acid sequences of isoforms 4 and 5. This site was infrequently lacking by the substitutions Ser32Gly as observed in the amino acid sequences of isoform 6.

Substitutions of amino acids were found mainly in the region βloop-3 and seatbelt of sardine Fshb. The seatbelt region is involved in heterodimer formation [1, 2]. On the other hand, the Fshb N-glycan is contributed to metabolic clearance and also recognized to be important for receptor binding and signaling [1]. The location of the N-glycosylation site is different among teleost species. Some fish species, such as African catfish (*Clarias gariepinus*), Atlantic halibut (*Hippoglossus hippoglossus*), and sablefish (*Anoplopoma fimbria*), lack this site [3–6]. To our best knowledge, the intraspecific variation of potential N-glycosylation site of Fshb has not been reported in teleost species. More work is necessary to identify the functional differences of sardine Fshb isoforms, such as the potency of receptor binding and steroid inducing.


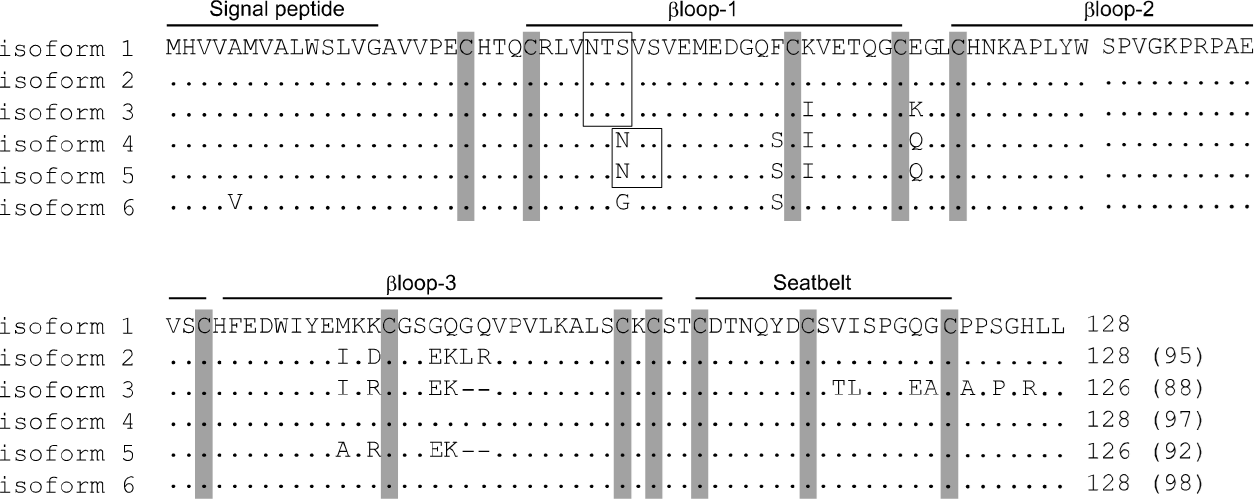


**Figure S10**

Sequence alignment of the amino acid sequences of Japanese sardine Fshb isoforms. Signal peptide prediction was performed with the SignalIP server (http://www.cbs.dtu.dk/services/SignalP/). Predictions of N-linked glycosylation were performed using the NetNGlyc 1.0 Server (http://www.cbs.dtu.dk/services/NetNGlyc/). Cysteine residues are shaded in gray. The putative amino acid glycosylation sites are boxed by solid lines. Numbers without parentheses show the amino acid number. Numbers in parentheses show amino acid identities between the main and each variant. GenBank accession numbers of Fshbs are as follows: isoform 1, LC545605; isoform 2, LC545606; isoform 3, LC545607; isoform 4, LC545608; isoform 5, LC545609; isoform 6, LC5456010.

**References**

1. Bousfield GR, Harvey DJ. Follicle-timulating hormone glycobiology. Endocrinology. 2019;160:1515–1535.

2. Swanson P, Dickey JT, Campbell B. Biochemistry and physiology of fish gonadotropins. Fish Physiol Biochem. 2003;28:53–59.

3. Guzmán JM, Luckenbach JA, Swanson P. Molecular characterization and quantification of sablefish (*Anoplopoma fimbria*) gonadotropins and their receptors: reproductive dysfunction in female captive broodstock. Gen Comp Endocrinol. 2013;193:37–47.

4. Imanaga Y, Nyuji M, Amano M, Takahashi A, Kitano H, Yamaguchi A, Matsuyama M. Characterization of gonadotropin-releasing hormone and gonadotropin in jack mackerel (*Trachurus japonicus*): comparative gene expression analysis with respect to reproductive dysfunction in captive and wild fish. Aquaculture. 2014;428–429:226–235.

5. Visher HF, Teves ACC, Mckermans JCM, van Dijk W, Schulz RW, Bogerd J. Cloning and spatiotemporal expression of the follicle-stimulating hormone β subunit complementary DNA in the African catfish (*Clarias gariepinus*). Biol Reprod. 2003;68: 1324–1332.

6. Weltzien FA, Kobayashi T, Andersson E, Norberg B, Andersen Ø. Molecular characterization and expression of FSHβ, LHβ, and common α-subunit in male Atlantic halibut (*Hippoglossus hippoglossus*). Gen Comp Endocrinol. 2003;131:87–96.
